# Author Correction: Single-cell transcriptomics uncovers EGFR signaling-mediated gastric progenitor cell differentiation in stomach homeostasis

**DOI:** 10.1038/s41467-023-41823-4

**Published:** 2023-10-17

**Authors:** Hitomi Takada, Yohei Sasagawa, Mika Yoshimura, Kaori Tanaka, Yoshimi Iwayama, Tetsutaro Hayashi, Ayako Isomura-Matoba, Itoshi Nikaido, Akira Kurisaki

**Affiliations:** 1 Laboratory of Stem Cell Technologies, Graduate School of Science and Technology, Nara Institute of Science and Technology, Takayama-cho, Ikoma, Nara Japan; 2 Laboratory for Bioinformatics Research, RIKEN Center for Biosystems Dynamics Research, Wako, Saitama Japan; 3 Department of Functional Genome Informatics, Biological Data Science, Medical Research Institute, Tokyo Medical and Dental University, Bunkyo, Tokyo Japan; 4 Master’s/Doctoral Program in Life Science Innovation (Bioinformatics), Degree Programs in Systems and Information Engineering, Graduate School of Science and Technology, University of Tsukuba, Tsukuba, Ibaraki Japan

**Keywords:** Adult stem cells, Stem-cell differentiation, Stem-cell niche

Correction to: *Nature Communications* 10.1038/s41467-023-39113-0, published online 29 June 2023

The original version of this Article contained an error in Fig. 5 and Supplementary Fig. 23. In the original version of Fig. 5f, the plot representing Muc5ac was given as a duplicate of that representing Gkn2. Additionally, the statement “the control data are also presented in Fig. 4b” was omitted in the legend of Fig. 5f. In the original version of Supplementary Figure 23g, an incorrect micrograph for control stained with SOX9 and DAPI was placed. This has been corrected in both the PDF and HTML versions of the Article.

### Supplementary information

Updated Supplementary Information

