# Supplementary material for: Single-cell transcriptomics uncovers EGFR signaling-mediated gastric progenitor cell differentiation in stomach homeostasis
Source: Nat Commun. 2023 Jun 29;14:3750. doi: 10.1038/s41467-023-39113-0 (PMC10310803; doi:10.1038/s41467-023-39113-0)
Supplement: Supplementary file 1 — Supplementary Information [file 41467_2023_39113_MOESM1_ESM.pdf]

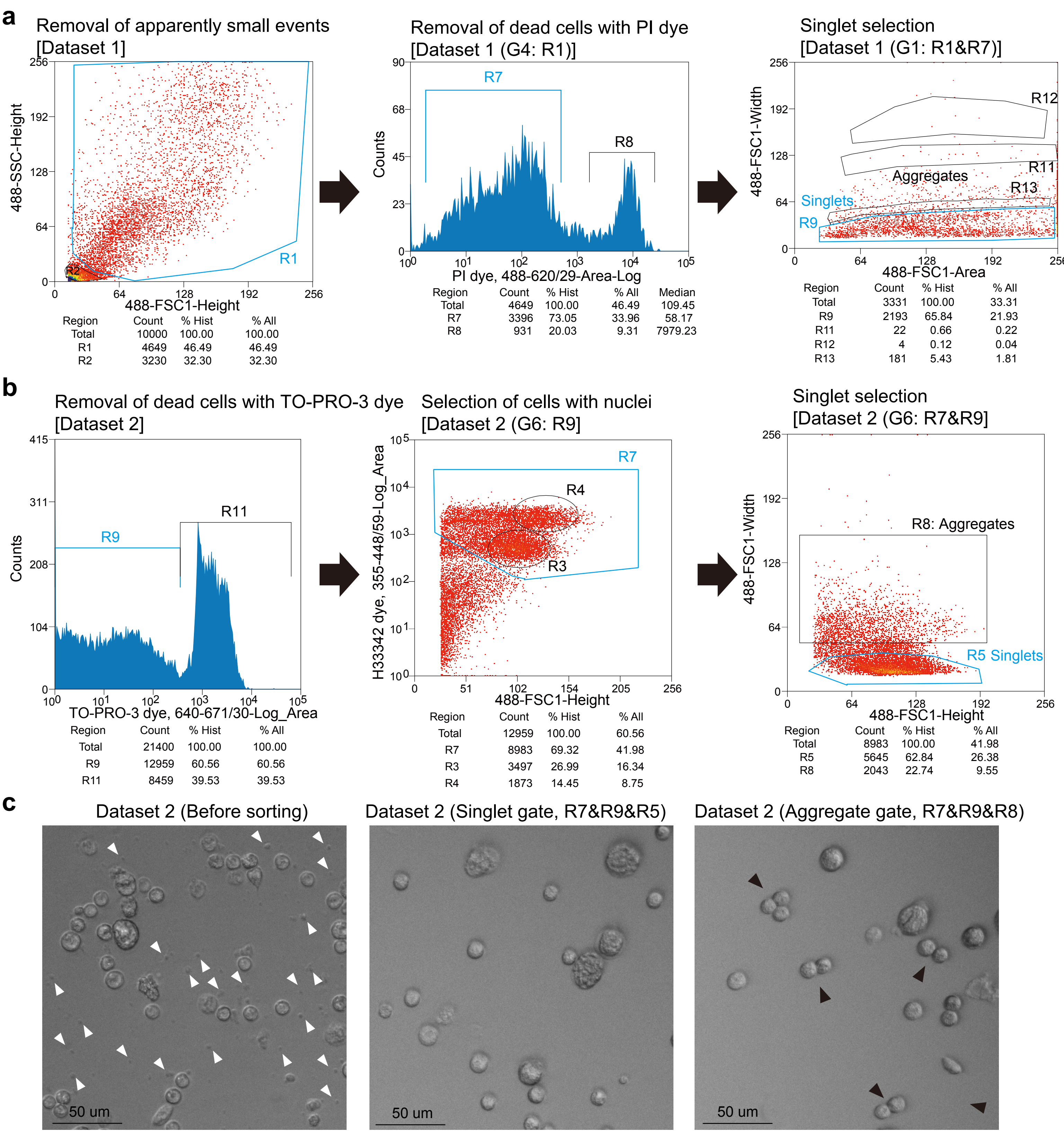

Supplementary Fig. 1 Single cell sorting of gastric cells. Related to Fig. 1

a Single cell soring of dataset 1. After the removal of small events (left, R1), dead cells were removed based on PI staining (middle, R7), and the cells in the singlet gate (right, R1&R7&R9) were sorted to 384 well plates.

b Single cell sorting of dataset 2. After the removal of dead cells by TO-PRO-3 staining (left, R9), the cells with nuclei were selected based on Hoechst 33342 (middle, R7), and cells in the singlet gate (right, R9&R7&R5) were sorted to 384 well plates.

c Phase contrast images of dissociated gastric cells used for single cell sorting in dataset 2. Before cell sorting (left), the cell sample contains debris (white arrowheads). The singlet gate R9&R7&R5 in (b) contains neither debris nor cell aggregates (middle). The aggregate gate R9&R7&R8 in (b) contains cell doublets, triplets (black arrowheads, right), and some larger cells. Scale bar, 50 μm. These images are representative of three images from one single cell sorting experiment.

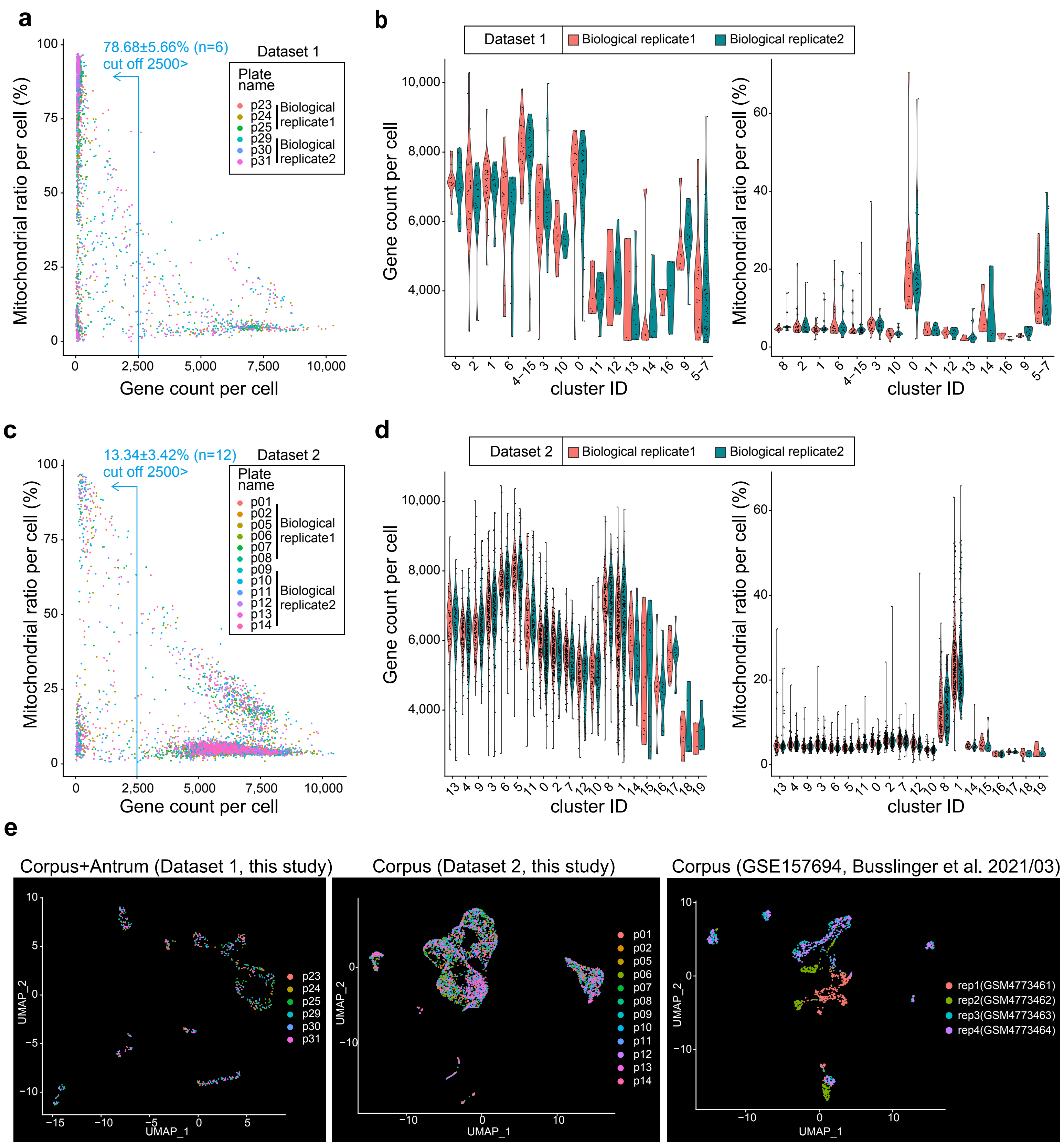
a
78.68±5.66% (n=6)
cut off 2500>
Dataset 1
Plate name
p23
p24
p25
Biological replicate1
p29
p30
p31
Biological replicate2
Mitochondrial ratio per cell (%)
Gene count per cell
b
Dataset 1
Biological replicate1
Biological replicate2
Gene count per cell
Mitochondrial ratio per cell (%)
cluster ID
c
13.34±3.42% (n=12)
cut off 2500>
Dataset 2
Plate name
p01
p02
p05
p06
p07
p08
Biological replicate1
p09
p10
p11
p12
p13
p14
Biological replicate2
Mitochondrial ratio per cell (%)
Gene count per cell
d
Dataset 2
Biological replicate1
Biological replicate2
Gene count per cell
Mitochondrial ratio per cell (%)
cluster ID
e
Corpus+Antrum (Dataset 1, this study)
Corpus (Dataset 2, this study)
Corpus (GSE157694, Busslinger et al. 2021/03)
UMAP_1
UMAP_2
rep1(GSM4773461)
rep2(GSM4773462)
rep3(GSM4773463)
rep4(GSM4773464)

Supplementary Fig. 2 Quality control of single-cell RNA-seq data. Related to Fig. 1

a Scatter plot showing gene count and mitochondrial ratio of all sequenced cells in dataset 1. The cells are colored by plate ID. The cells with less than 2,500 gene counts were removed as low-quality cells.

b Violin plots showing gene count and mitochondrial ratio of the cells that passed quality control in each cluster. Two biological replicates prepared by different operators were shown in red and green.

c Scatter plot showing gene count and mitochondrial ratio of all sequenced cells in dataset 2. The cells are colored by plate ID. The cells with less than 2,500 gene counts were removed as low-quality cells.

d Violin plots showing gene count and mitochondrial ratio of the cells that passed quality control in each cluster. Two biological replicates prepared by different operators were shown in red and green. **e** UMAP of gastric cells in dataset 1 (left, our study), dataset 2 (middle, our study), and the previously published dataset (right, GSE157694). In our study, the cells were colored by plate ID. In the previously published data, the cells were colored by plates. Note that, in our study, the cells from six plates (dataset 1, two biological replicates) or twelve plates (dataset 2, two biological replicates) were clustered together, suggesting that our data is not affected by technical noise.

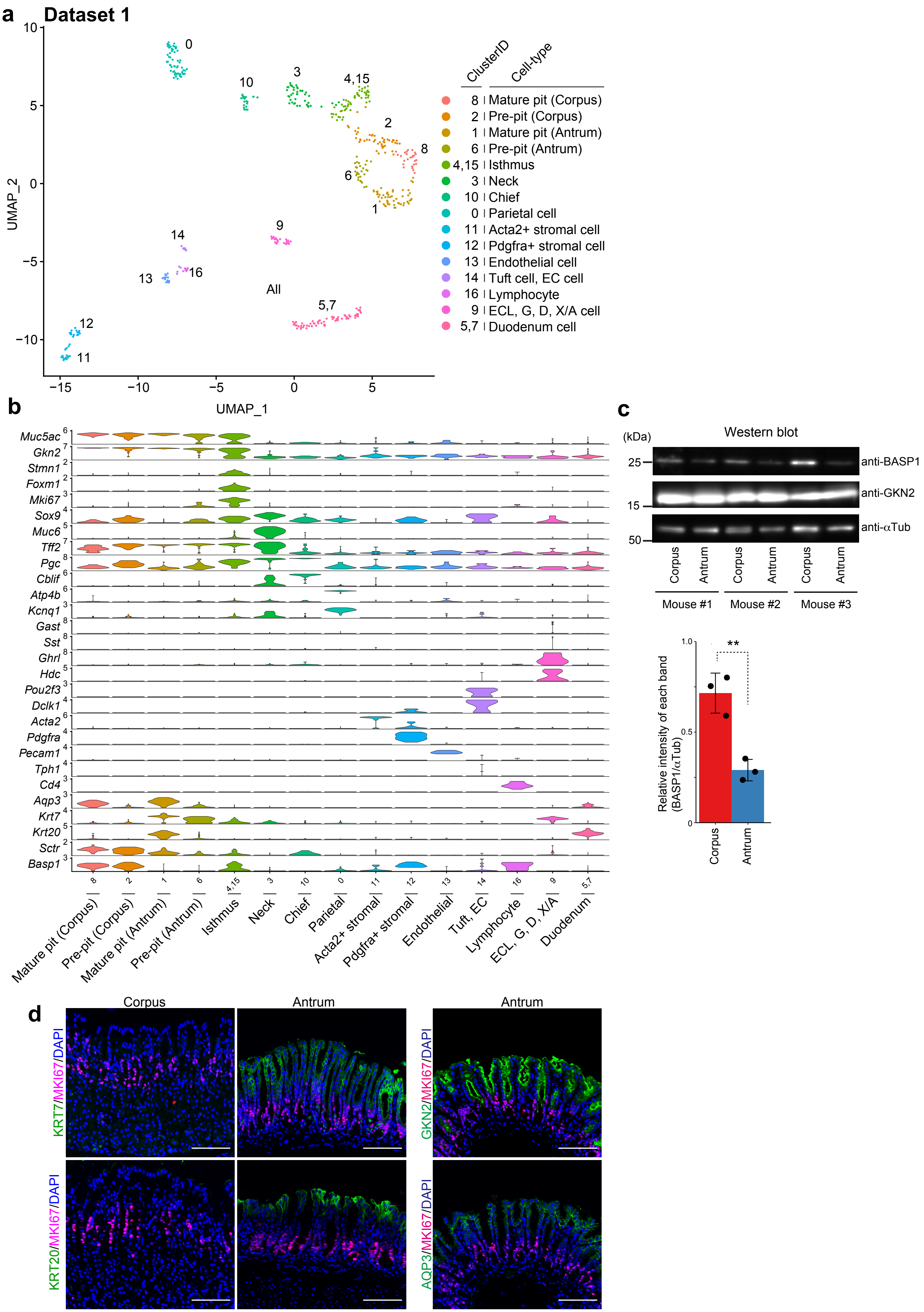
a
Dataset 1
UMAP_1
UMAP_2
All
ClusterID
Cell-type
8 Mature pit (Corpus)
2 Pre-pit (Corpus)
1 Mature pit (Antrum)
6 Pre-pit (Antrum)
4,15 Isthmus
3 Neck
10 Chief
0 Parietal cell
11 Acta2+ stromal cell
12 Pdgfra+ stromal cell
13 Endothelial cell
14 Tuft cell, EC cell
16 Lymphocyte
9 ECL, G, D, X/A cell
5,7 Duodenum cell
b
Muc5ac
Gkn2
Stmn1
Foxm1
Mki67
Sox9
Muc6
Tff2
Pgc
Cblif
Atp4b
Kcnq1
Gast
Sst
Ghrl
Hdc
Pou2f3
Dclk1
Acta2
Pdgfra
Pecam1
Tph1
Cd4
Aqp3
Krt7
Krt20
Sctr
Basp1
Mature pit (Corpus)
Pre-pit (Corpus)
Mature pit (Antrum)
Pre-pit (Antrum)
Isthmus
Neck
Chief
Parietal
Acta2+ stromal
Pdgfra+ stromal
Endothelial
Tuft, EC
Lymphocyte
ECL, G, D, X/A
Duodenum
c
Western blot
(kDa)
anti-BASP1
anti-GKN2
anti-αTub
Corpus
Antrum
Mouse #1
Mouse #2
Mouse #3
**
Relative intensity of each band
(BASP1/αTub)
d
Corpus
Antrum
KRT7/MKI67/DAPI
KRT20/MKI67/DAPI
GKN2/MKI67/DAPI
AQP3/MKI67/DAPI

Supplementary Fig. 3. Single-cell analysis of corpus and antrum gastric units (dataset 1).

a UMAP visualization of the cells isolated from the corpus and antrum gastric units (dataset 1). Cells are colored according to the clustering results.

b Violin plots of dataset 1 showing the expression of known marker genes in each cluster.

c Western blotting showing the expression of BASP1 (top), GKN2 (middle), and α-Tubulin (bottom) in corpus and antrum glands isolated from three different mice. Data are presented as mean relative expression ± SD. Significance was calculated by two-tailed Student's t-tests (***p* = 0.0042). Source data are provided as a Source Data file.

d Immunofluorescence staining of adult mouse corpus and antrum tissue with KRT7 (green), KRT20 (green), MKI67 (red), and DAPI (blue). Scale bar, 100 μm. The images of KRT20, GKN2, and AQP3 staining are representative of three independent experiments. The images of KRT7 staining are representative of two independent experiments.

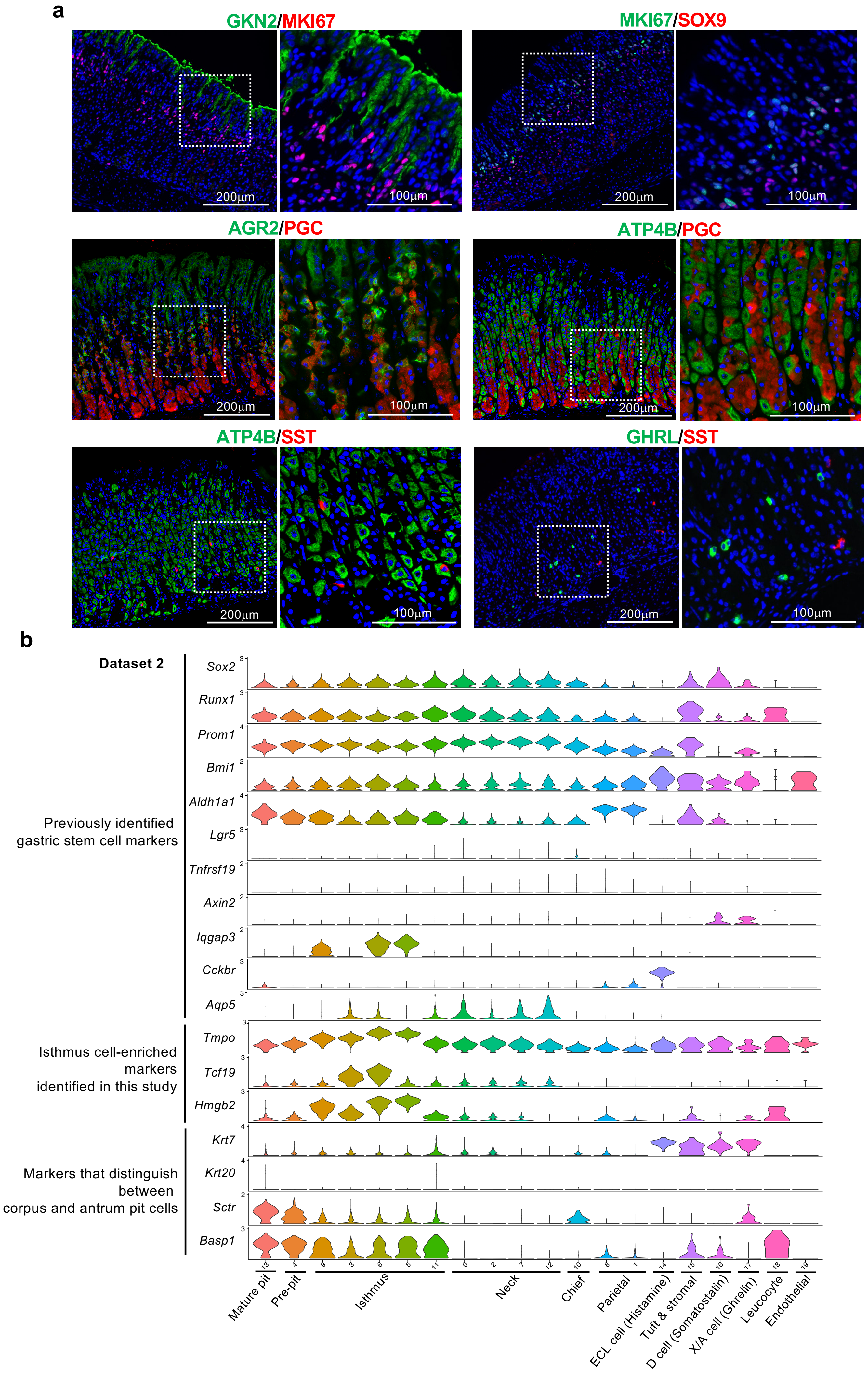
a
GKN2/MKI67
200μm
100μm
MKI67/SOX9
200μm
100μm
AGR2/PGC
200μm
100μm
ATP4B/PGC
200μm
100μm
ATP4B/SST
200μm
100μm
GHRL/SST
200μm
100μm
b
Dataset 2
Previously identified gastric stem cell markers
Isthmus cell-enriched markers identified in this study
Markers that distinguish between corpus and antrum pit cells
Sox2
Runx1
Prom1
Bmi1
Aldh1a1
Lgr5
Tnfrsf19
Axin2
Iqgap3
Cckbr
Aqp5
Tmpo
Tcf19
Hmgb2
Krt7
Krt20
Sctr
Basp1
13
4
9
3
6
5
11
0
2
7
12
10
8
1
14
15
16
17
18
19
Mature pit
Pre-pit
Isthmus
Neck
Chief
Parietal
ECL cell (Histamine)
Tuft & stromal
D cell (Somatostatin)
X/A cell (Ghrelin)
Leucocyte
Endothelial

Supplementary Fig. 4. Expression pattern of various gastric epithelial cell markers in adult mouse corpus tissue. Related to Fig. 1.

a Immunofluorescence staining of the corpus tissues with cell type-specific markers. High magnification images of the dotted squares are shown on the right side. All images are representative of two independent experiments.

b Violin plots of dataset 2 showing the expression of previously identified gastric stem cell markers, isthmus progenitor cell-enriched markers identified in this study, and the markers that distinguish between corpus and antrum pit cells.

Dataset 2

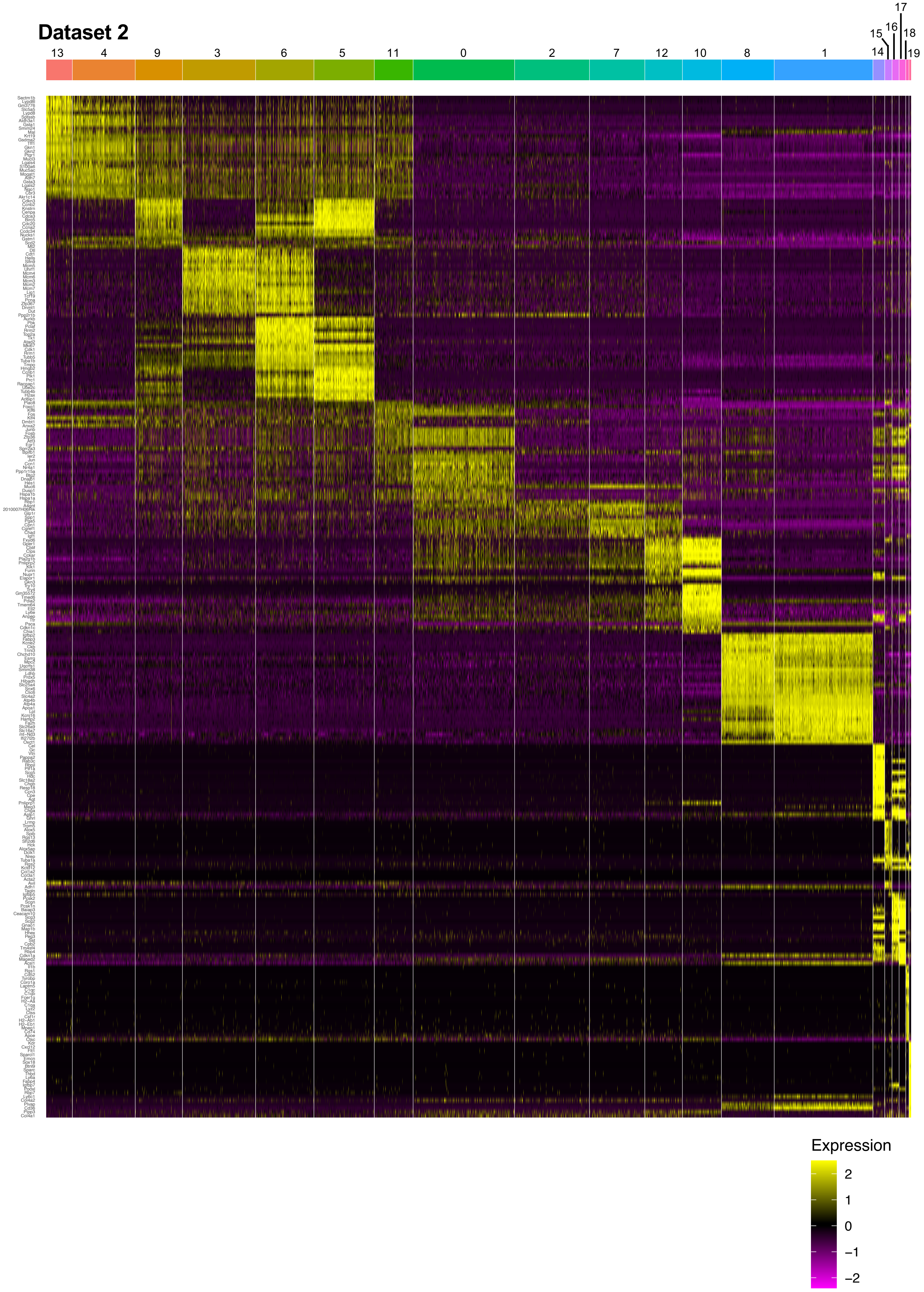

Supplementary Fig. 5. Cell type-specific markers identified in dataset 2. Related to Fig. 1

Heatmap showing the expression of cell type-specific marker genes in individual cells. Differentially expressed genes with LogFC > 0.75 were used to generate this heatmap.

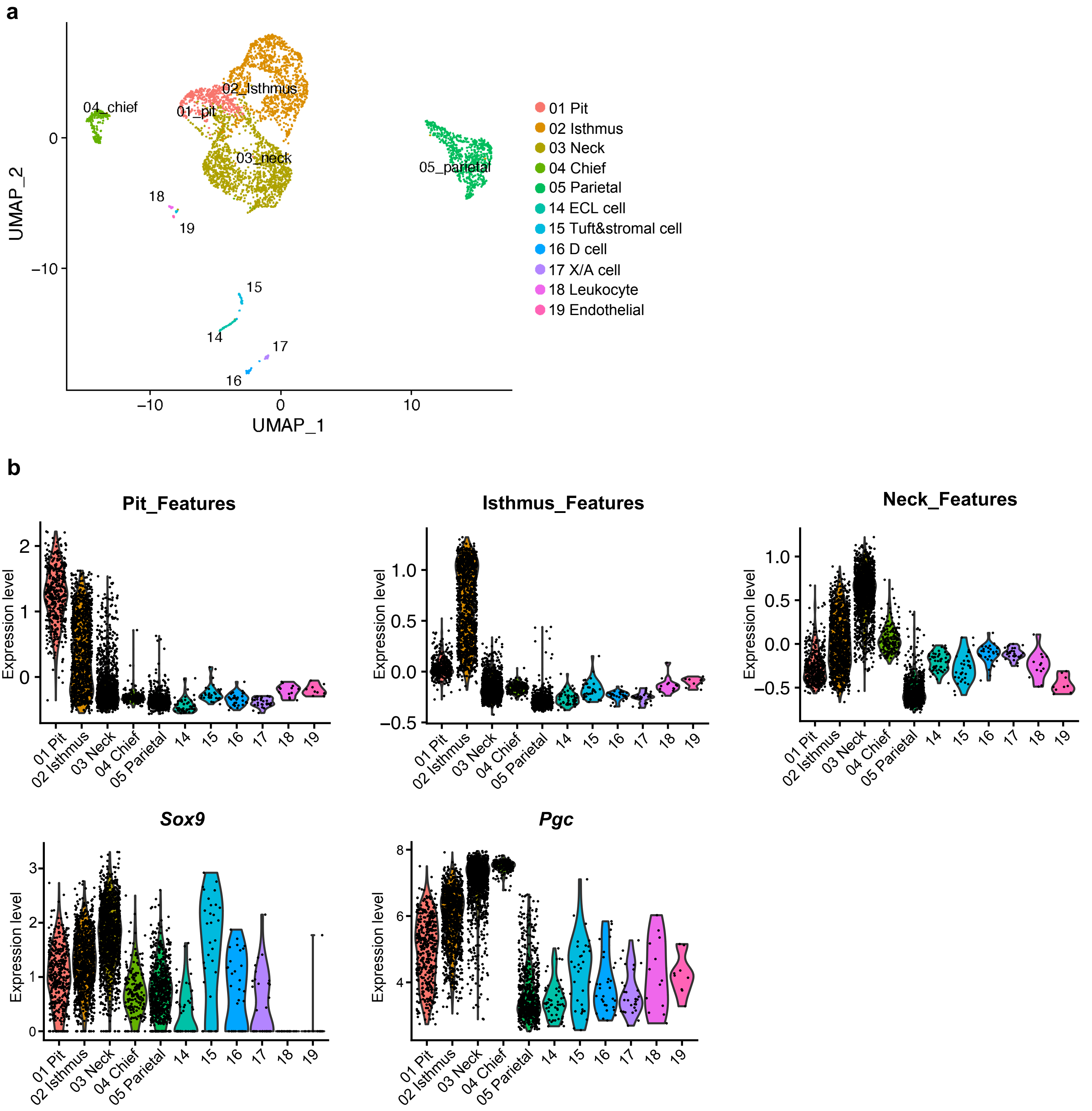

Supplementary Fig. 6. Expression of Sox9 and Pgc in combined clusters. Related to Fig. 1

a UMAP visualization of the cells identified in dataset 2. The multiple clusters of same cell types were combined into one cluster.

b Top: violin plots showing the expression of top 50 genes enriched in pit cells (Pit_Features), in isthmus progenitor cells (Isthmus_Features), and in neck cells (Neck_Features). Bottom: violin plots showing the expression of *Sox9* and *Pgc*.

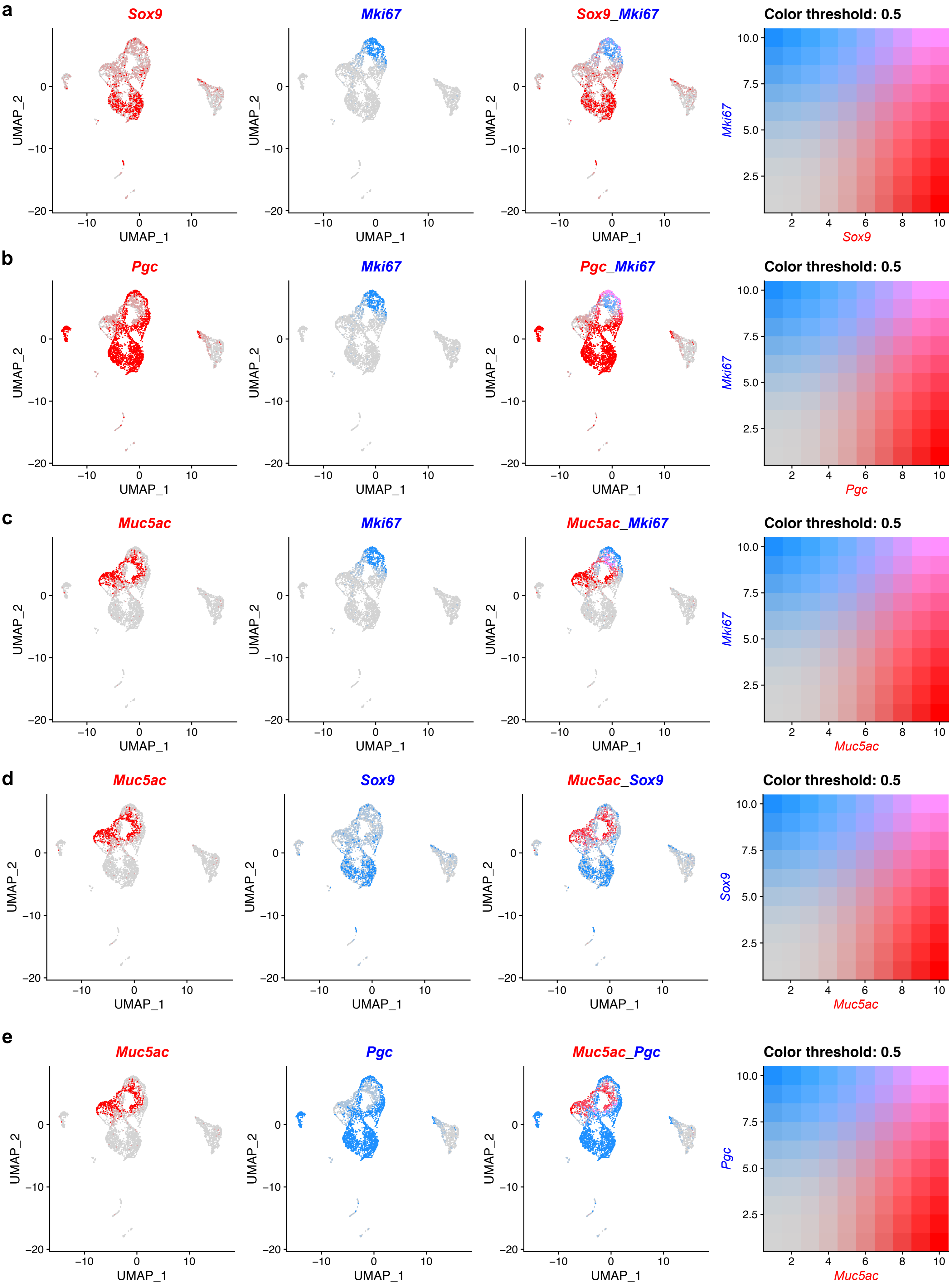
a
Sox9
Mki67
Sox9_Mki67
Color threshold: 0.5
b
Pgc
Mki67
Pgc_Mki67
Color threshold: 0.5
c
Muc5ac
Mki67
Muc5ac_Mki67
Color threshold: 0.5
d
Muc5ac
Sox9
Muc5ac_Sox9
Color threshold: 0.5
e
Muc5ac
Pgc
Muc5ac_Pgc
Color threshold: 0.5
UMAP_1
UMAP_2

Supplementary Fig. 7. Comparison of the expression profiles of *Muc5ac, Mki67, Sox9,* and *Pgc.* Related to Fig. 1

UMAP of gastric cells colored by the expression level of *Sox9* and *Mki67* (**a**), *Pgc* and *Mki67* (**b**), *Muc5ac* and *Mki67* (**c**), *Muc5ac* and *Sox9* (**d**), and *Muc5ac* and *Pgc* (**e**).

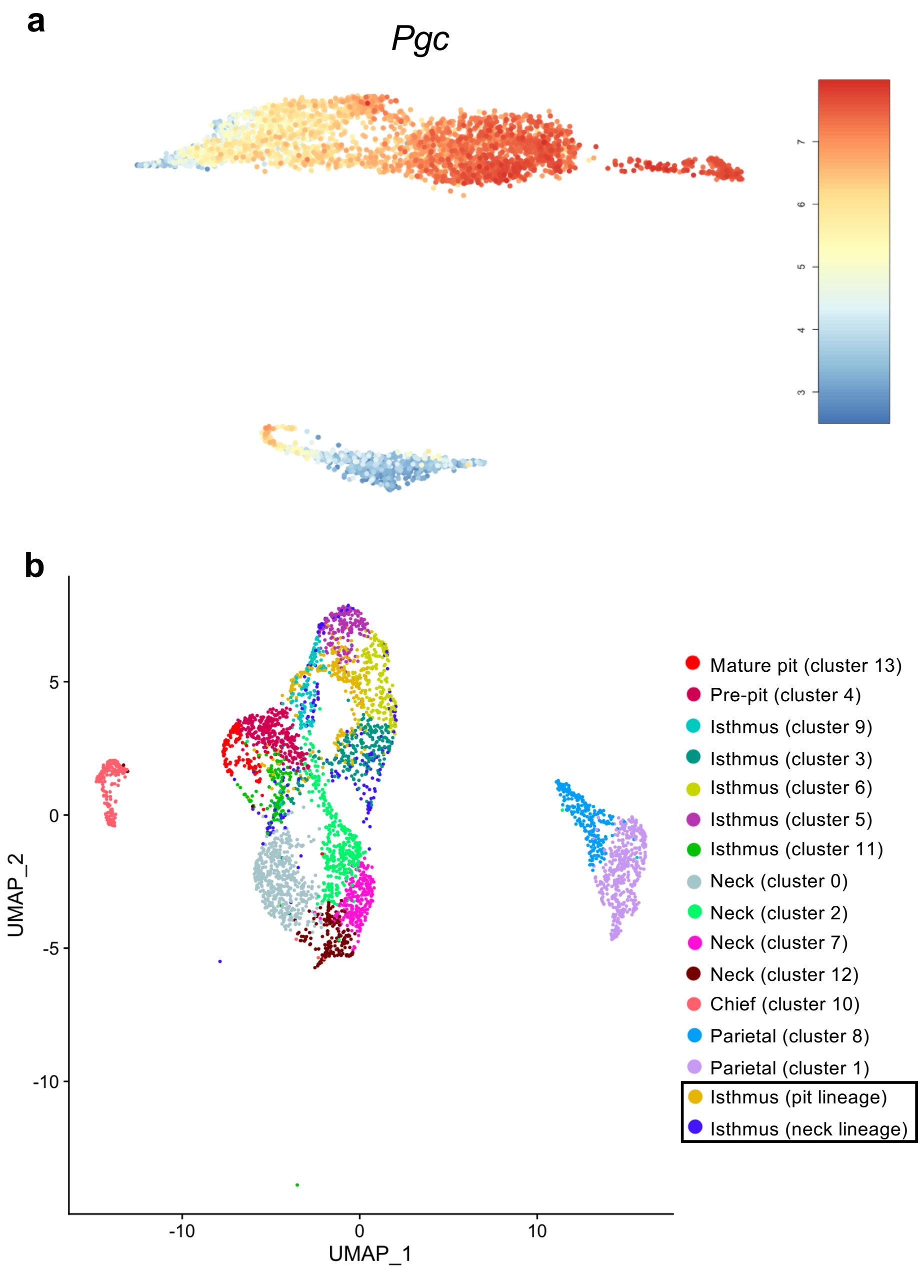

Supplementary Fig. 8. Psuedotime analysis of major corpus epithelial cells identified in dataset 2. Related to Fig. 2.

a The expression level of Pgc along differentiation trajectories (dataset 2).

b UMAP visualization of isthmus progenitor cells identified in pit cell or neck cell lineage by FateID analysis. Note that isthmus progenitor cells used for pit cell (yellow) or neck cell (blue) lineage by FateID were merged with isthmus progenitor cells with high pit cell feature or high neck cell feature identified in Fig. 1f, supporting the reliability of FateID analysis.

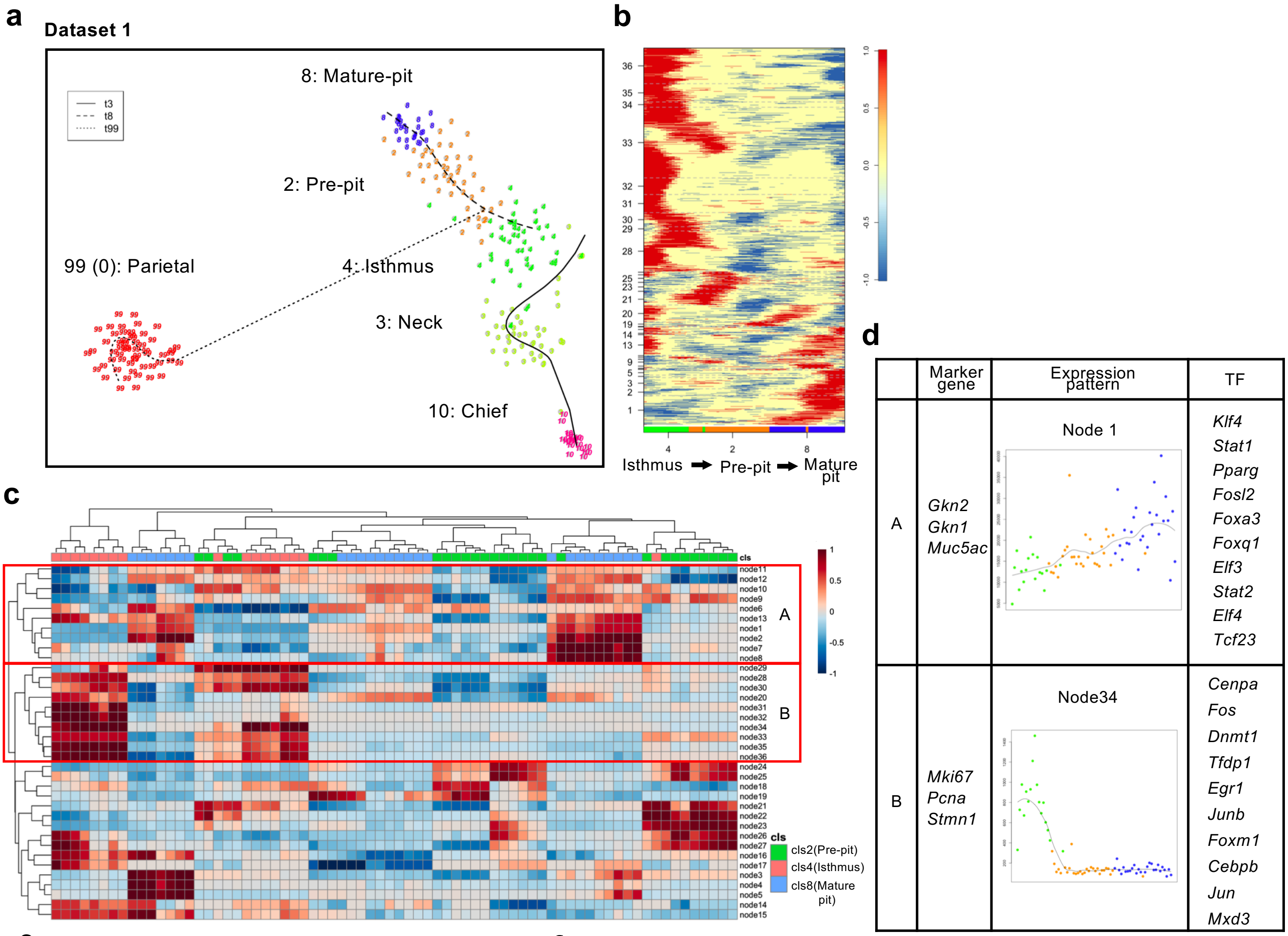

e

Group A (Upregulation)

Pathways

Term	adj. p-value
EGFR1 signaling pathway	9.4047E-05
Triacylglyceride synthesis	0.00054875
Alpha 6 beta 4 integrin signaling pathway	0.0007502
Sphingolipid metabolism overview	0.00614722
Mitochondrial long chain fatty acid beta-oxidation	0.00657058
Fatty acid beta-oxidation	0.00845411
Sphingolipid metabolism (integrated pathway)	0.00926181
Eicosanoid metabolism via cyclooxygenases (COX)	0.01087412
Alzheimer's disease	0.01483459
IL-3 signaling pathway	0.01719836

GO (Biological Pathway)

Term	adj. p-value
organonitrogen compound biosynthetic process	1.02E-05
sphingolipid biosynthetic process	2.67E-05
membrane lipid biosynthetic process	3.44E-05
membrane organization	4.40E-05
multivesicular body organization	2.58E-04

f

Group B (Downregulation)

Pathways

Term	adj. p-value
mRNA processing	9.86E-39
TNF-alpha NF-kB signaling pathway	1.47E-14
DNA replication	9.37E-14
Cytoplasmic ribosomal proteins	2.13E-12
G1 to S cell cycle control	1.85E-10
Proteasome degradation	3.01E-09
Electron transport chain	1.70E-08
Eukaryotic transcription initiation	2.43E-08
Estrogen signaling	4.2702E-06
TCA cycle	0.00020323

GO (Biological Pathway)

Term	adj. p-value
mRNA processing	3.38E-67
mRNA splicing, via spliceosome	2.62E-65
RNA splicing, via transesterification reactions with bulged adenosine as nucleophile	3.08E-61
gene expression	6.26E-57
cellular macromolecule biosynthetic process	4.04E-52

Supplementary Fig. 9. Characterization of the pit cell differentiation process in dataset 1. Related to Fig.2.

a Pseudotemporal ordering of major corpus epithelial cells identified in dataset 1. Principal curves are shown for mature pit cells (t8, heavy dotted line), neck cells (t3, solid line), and parietal cells (t99, fine dotted line).

b Self-organizing map of binarized pseudotemporal expression profiles along the pit cell differentiation trajectory. The x-axis indicates the cells involved in the pit cell lineage that are colored according to cell types and the y-axis indicates the nodes.

c Hierarchical clustering of co-expression nodes in (b). The nodes in group A and B show upregulation and downregulation patterns of the genes in the pit cell differentiation trajectory, respectively. The x-axis indicates the cells involved in the pit cell lineage that are colored according to cell types and the y-axis indicates the nodes. The x-axis in (c) is not same as that of (b) due to the new hierarchical clustering of the cells. The colors for each cell type are shown on the right bottom corner.

d Characteristics of group A and B defined in (c). Previously reported marker genes in each group are shown. Average pseudotemporal expression profile of the representative node is shown in gray line. The x-axis indicates pseudotime from isthmus progenitor cells to pit cells and the y-axis indicates expression level. Colors of each dot represent cell types, which corresponds to colors used in (a). Among the TFs involved in group A and B, the top 10 TFs specifically expressed in pit cells and isthmus progenitor cells (LogFC > 0.25) are shown, respectively.

e and f Characterization of the pseudotime-dependent genes included in group A (e) and group B (f). Upper panel, pathway enrichment analysis of the pseudotime-dependent genes. The top ten pathways with significant enrichment (adjusted p-value < 0.05) are listed. Lower panel, GO analysis of the pseudotime-dependent genes. The top five terms with significant enrichment (adjusted p-value < 0.05) are listed. Adjusted p-value was determined using one-tailed Fisher's exact test with g:SCS method in g:Profiler[36] (pathways) and Fisher's exact test with Benjamini-Hochberg adjustment in Enrichr[37] (GO).

a

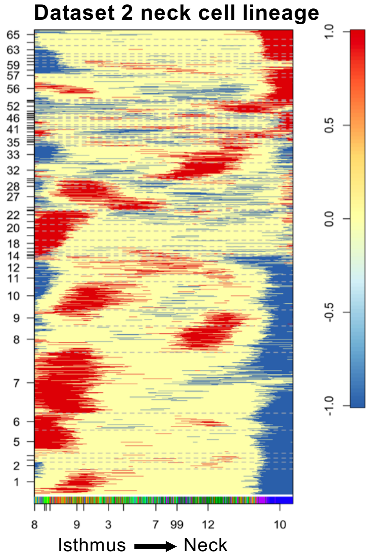

b

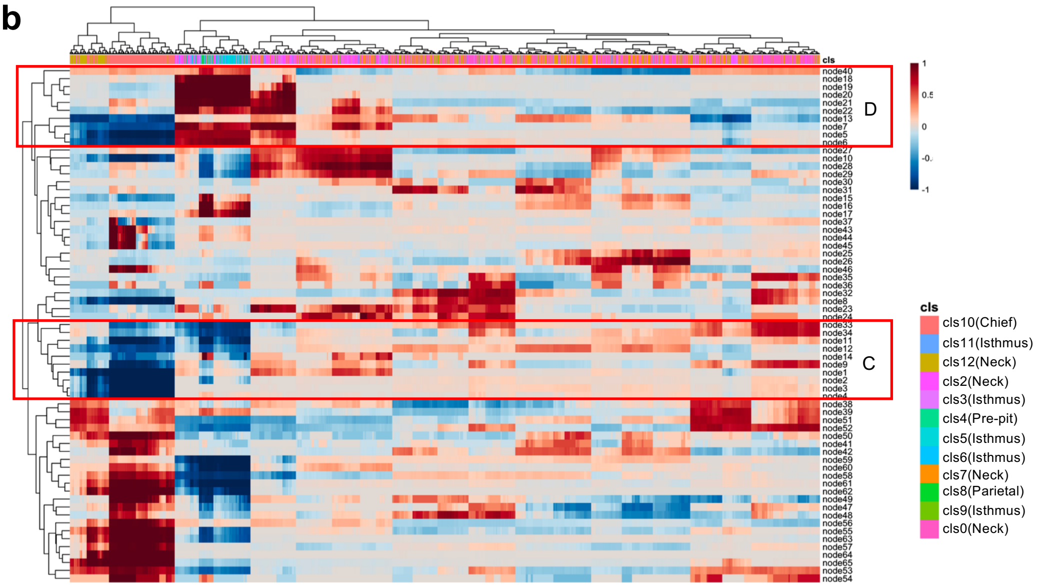

c

	Marker gene	Expression pattern	TF
C	*Sox9* *Muc6*	Node33 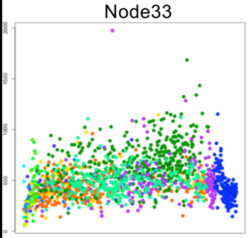	*Hes1* *Pbx1* *Spdef* *Sox9* *Klf5* *Mxd4* *Isl1* *Sox4* *Mecom* *Foxa1*
D	*Mki67* *Pcna* *Stmn1*	Node18 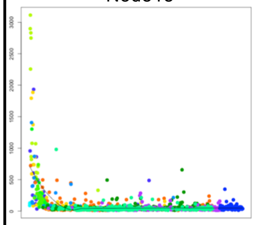	*Cenpa* *Dnmt1* *Tfdp1* *Klf4* *Foxm1* *Mxd3* *Terf1* *Maz* *Ybx3* *Lin54*

d

Group C (Upregulation)

Pathways

Term	adj. p-value
Kit receptor signaling pathway	0.0029467
Fatty acid biosynthesis	0.00316366
IL-7 signaling pathway	0.00600176
IL-5 signaling pathway	0.01178
IL-6 signaling pathway	0.01562132
Wnt signaling pathway (NetPath)	0.03008174

GO (Cellular Component)

Term	adj. p-value
lysosome	4.80E-08
lysosomal membrane	7.82E-05
lytic vacuole membrane	1.07E-04
lytic vacuole membrane	5.46E-04
lysosomal lumen	0.00198682

e

Group D (Downregulation)

Pathways

Term	adj. p-value
mRNA processing	9.04E-29
Cytoplasmic ribosomal proteins	7.10E-20
DNA replication	6.86E-15
Electron transport chain	3.33E-10
G1 to S cell cycle control	3.69E-10
Proteasome degradation	6.94E-08
TNF-alpha NF-kB signaling pathway	7.02E-07
Cholesterol biosynthesis	9.2308E-05
Mismatch repair	0.00034072
Oxidative phosphorylation	0.0007799

GO (Biological Process)

Term	adj. p-value
cellular macromolecule biosynthetic process	1.29E-54
rRNA processing	8.83E-50
ribosome biogenesis	8.83E-50
ncRNA processing	8.27E-48
rRNA metabolic process	6.23E-43

Supplementary Fig. 10. Characterization of the neck cell differentiation process in dataset 2. Related to Fig.2.

a Self-organizing map of binarized pseudotemporal expression profiles along the neck cell differentiation trajectory. The x-axis indicates the cells involved in the neck cell lineage that are colored according to cell types and the y-axis indicates the nodes.

b Hierarchical clustering of co-expression nodes in (b). The nodes in group C and D show upregulation and downregulation patterns of the genes in the neck cell differentiation trajectory, respectively. The x-axis indicates the cells involved in the neck cell lineage that are colored according to cell types and the y-axis indicates the nodes. The x-axis in (b) is not same as that of (a) because of the new hierarchical clustering of the cells. The colors for each cell type are shown on the right bottom corner.

c Characteristics of group C and D identified in (b). Marker genes in each group are shown. Average pseudotemporal expression profile of the representative node is shown in black line. The x-axis indicates pseudotime from isthmus progenitor cells to neck cells and the y-axis indicates expression level. Colors in each dot represent cell types, which corresponds to colors used in Fig. 2a. Among the TFs involved in group C and D, the top 10 TFs specifically expressed in neck cells and isthmus progenitor cells (LogFC > 0.25) are shown here, respectively.

d and e Characterization of the pseudotime-dependent genes included in group C (d) and group D (e). Upper panel, pathway enrichment analysis of the pseudotime-dependent genes. The six pathways and the top ten pathways with significant enrichment (adjusted *p*-value < 0.05) are listed in group C and D, respectively. Lower panel, GO analysis of the pseudotime-dependent genes. The top five terms with significant enrichment (adjusted *p*-value < 0.05) are listed. Adjusted *p*-value was determined using one-tailed Fisher's exact test with g:SCS method in g:Profiler[36] (pathways) and Fisher's exact test with Benjamini-Hochberg adjustment in Enrichr[37] (GO).

a

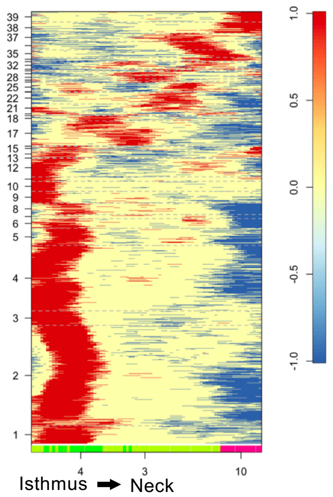

b

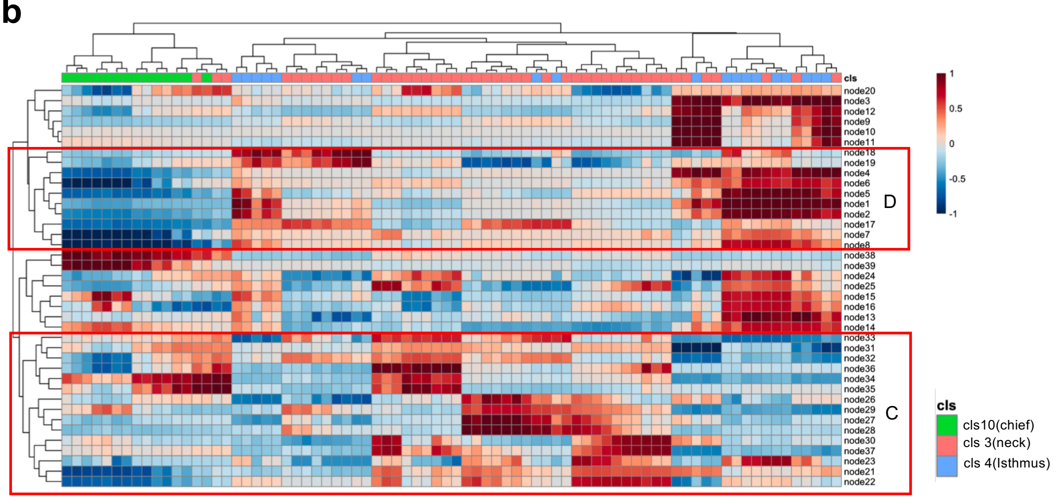

c

	Marker gene	Expression pattern	TF
C	*Sox9*<br>*Muc6*	Node36<br>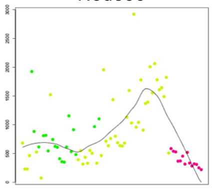	*Hes1*<br>*Pbx1*<br>*Spdef*<br>*Sox9*<br>*Irf1*<br>*Nfil3*<br>*Mxd4*<br>*Isl1*<br>*Tcf12*<br>*Mecom*
D	*Mki67*<br>*Pcna*<br>*Stmn1*	Node2<br>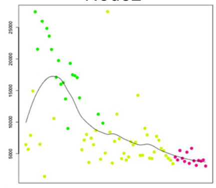	*Cenpa*<br>*Klf6*<br>*Dnmt1*<br>*Tfdp1*<br>*Foxq1*<br>*Klf4*<br>*Foxm1*<br>*Cebpb*<br>*Mxd3*<br>*Terf1*

d

Group C (Upregulation)

Pathways

Term	adj. p-value
Insulin signaling	0.00133206
Focal adhesion: PI3K-Akt-mTOR signaling pathway	0.00230234
G protein signaling pathways	0.01157589
MicroRNAs in cardiomyocyte hypertrophy	0.04435215

GO (Cellular Component)

Term	adj. p-value
lysosome	4.25E-10
bounding membrane of organelle	2.96E-08
lytic vacuole	8.15E-08
lysosomal lumen	1.13E-07
lytic vacuole membrane	1.22E-06

e

Group D (Downregulation)

Pathways

Term	adj. p-value
mRNA processing	3.84E-40
TNF-alpha NF-kB signaling pathway	7.10E-14
Cytoplasmic ribosomal proteins	1.09E-13
DNA replication	8.74E-12
Proteasome degradation	3.85E-10
Electron transport chain	1.11E-09
G1 to S cell cycle control	6.17E-09
Eukaryotic transcription initiation	4.39E-08
TCA cycle	6.4025E-05
Estrogen signaling	0.00026762

GO (Cellular Component)

Term	adj. p-value
mRNA processing	4.09E-54
mRNA splicing, via spliceosome	5.60E-53
RNA splicing, via transesterification reactions with bulged adenosine as nucleophile	3.58E-50
gene expression	3.08E-46
cellular macromolecule biosynthetic process	2.85E-41

Supplementary Fig. 11. Characterization of the neck cell differentiation process in dataset 1. Related to Fig.2.

a Self-organizing map of binarized pseudotemporal expression profiles along the neck cell differentiation trajectory. The x-axis indicates the cells involved in the neck cell lineage that are colored according to cell types and the y-axis indicates the nodes.

b Hierarchical clustering of co-expression nodes in (b). The nodes in group C and D show upregulation and downregulation patterns of the genes in the neck cell differentiation trajectory, respectively. The x-axis indicates the cells involved in the neck cell lineage that are colored according to cell types and the y-axis indicates the nodes. The x-axis in (b) is not same as that of (a) because of the new hierarchical clustering of the cells. The colors for each cell type are shown on the right bottom corner.

c Characteristics of group C and D identified in (b). Previously reported marker genes in each group are shown. Average pseudotemporal expression profile of the representative node is shown in gray line. The x-axis indicates pseudotime from isthmus progenitor cells to neck cells and the y-axis indicates expression level. Colors in each dot represent cell types, which corresponds to colors used in Supplementary Fig. 7a. Among the TFs involved in group C and D, the top 10 transcription factors specifically expressed in neck cells and isthmus progenitor cells (LogFC > 0.25) are shown, respectively.

d and e Characterization of the pseudotime-dependent genes included in group C (d) and group D (e). Upper panel, pathway enrichment analysis of the pseudotime-dependent genes. The four pathways and the top ten pathways with significant enrichment (adjusted *p*-value < 0.05) are listed in group C and D, respectively. Lower panel, GO analysis of the pseudotime-dependent genes. The top five terms with significant enrichment (adjusted *p*-value < 0.05) are listed. Adjusted *p*-value was determined using one-tailed Fisher's exact test with g:SCS method in g:Profiler[36] (pathways) and Fisher's exact test with Benjamini-Hochberg adjustment in Enrichr[37] (GO).

a

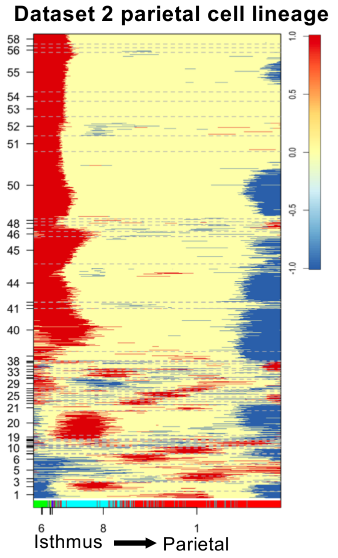

b

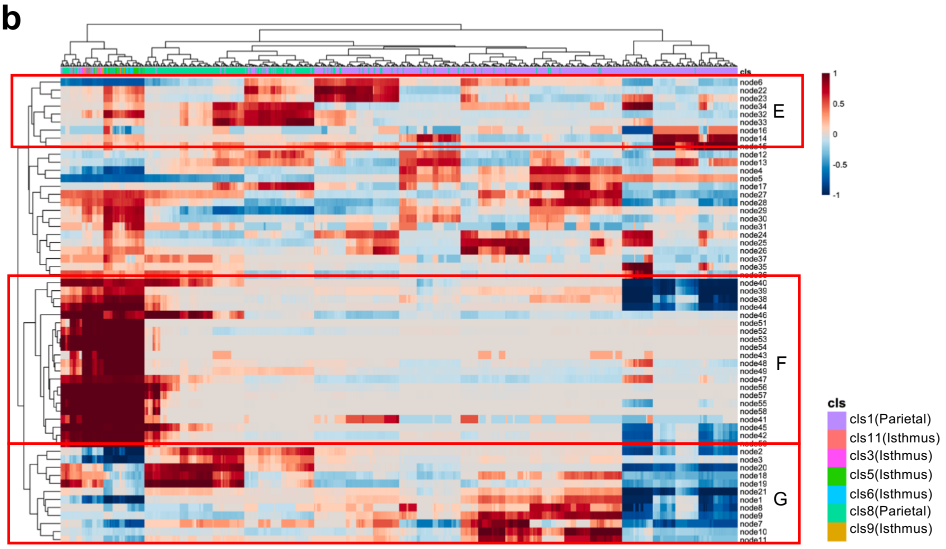

c

	Marker gene	Expression pattern	TF
E	*Atp4b* *Kcnq1*	Node6 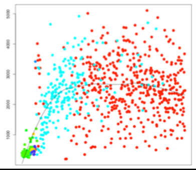	
F	*Mki67* *Pcna* *Stmn1*	Node51 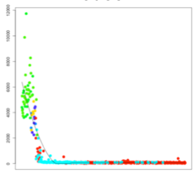	*Cenpa* *Klf6* *Fos* *Dnmt1* *Fosb* *Tfdp1* *Atf3* *Foxq1* *Klf4* *Egr1*
G		Node20 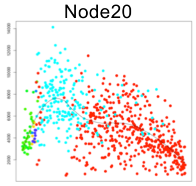	*Esrrg* *Chchd3* *Esrra* *Ybx2* *Mitf* *Nacc2* *Cxxc5* *Arhgap35* *Snapc5* *Mbnl2*

d

Group F (Downregulation)

Pathways

Term	adj. p-value
mRNA processing	3.60E-47
TNF-alpha NF-kB signaling pathway	1.75E-27
Estrogen signaling	5.50E-15
Cytoplasmic ribosomal proteins	2.63E-12
G1 to S cell cycle control	4.52E-12
EGFR1 signaling pathway	7.13E-12
Eukaryotic transcription initiation	1.46E-11
PluriNetWork: mechanisms associated with pluripotency	2.71E-10
DNA replication	3.17E-10
Translation factors	6.29E-09
Proteasome degradation	1.50E-07
IL-6 signaling pathway	0.0001212
FAS pathway and stress induction of HSP regulation	0.00016859
Wnt signaling pathway (NetPath)	0.00017451
Apoptosis	0.0004891
IL-7 signaling pathway	0.00052432
Delta-Notch signaling pathway	0.00139537
IL-5 signaling pathway	0.00390991
IL-3 signaling pathway	0.00496692
Toll-like receptor signaling	0.00849418

e

Group G (Transient upregulation)

Pathways

Term	adj. p-value
Electron transport chain	1.34E-45
Oxidative phosphorylation	1.41E-27
TCA cycle	3.41E-17
Fatty acid beta-oxidation	1.44E-09
Amino acid metabolism	1.17E-07
Glycolysis and gluconeogenesis	3.2851E-06
Mitochondrial long chain fatty acid beta-oxidation	5.642E-06
Fatty acid biosynthesis	0.01778986
Insulin signaling	0.0186317
Arachidonate epoxygenase / epoxide hydrolase	0.03885906

GO (Biological Pathway)

Term	adj. p-value
aerobic electron transport chain	7.30E-53
mitochondrial ATP synthesis coupled electron transport	2.63E-52
mitochondrial respiratory chain complex assembly	6.58E-37
mitochondrial electron transport, NADH to ubiquinone	3.64E-36
mitochondrial respiratory chain complex I assembly	2.68E-33

Supplementary Fig. 12. Characterization of the parietal cell differentiation process in dataset 2. Related to Fig.2.

a Self-organizing map of binarized pseudotemporal expression profiles along the parietal cell differentiation trajectory. The x-axis indicates the cells involved in the parietal cell lineage that are colored according to cell types and the y-axis indicates the nodes.

b Hierarchical clustering of co-expression nodes in (a). The nodes in group E, F, and G show upregulation, downregulation, and transient upregulation patterns of the genes in the parietal cell differentiation trajectory, respectively. The x-axis indicates the cells involved in the parietal cell lineage that are colored according to cell types and the y-axis indicates the nodes. The x-axis in (b) is not same as that of (a) because of the new hierarchical clustering of the cells. The colors for each cell type are shown on the right bottom corner.

c Characteristics of group E, F, and G identified in (b). Previously reported marker genes in each group are shown. Average pseudotemporal expression profile of the representative node is shown in black line. The x-axis indicates pseudotime from isthmus progenitor cells to parietal cells and the y-axis indicates expression level. Colors of each dot represent cell types, which corresponds to colors used in Fig. 2a. Among the TFs involved in group E, F, and G, the top 10 TFs specifically expressed in parietal cells and isthmus progenitor cells (LogFC > 0.25) are shown, respectively.

d and e Characterization of the pseudotime-dependent genes included in group F (d) and group G (e). Upper panel, pathway enrichment analysis of the pseudotime-dependent genes. The top 20 and the top 10 pathways with significant enrichment (adjusted *p*-value < 0.05) are listed in group F and G, respectively. Lower panel, GO analysis of the pseudotime-dependent genes. The top five terms with significant enrichment (adjusted *p*-value < 0.05) are listed. Adjusted *p*-value was determined using one-tailed Fisher's exact test with g:SCS method in g:Profiler[36] (pathways) and Fisher's exact test with Benjamini-Hochberg adjustment in Enrichr[37] (GO).

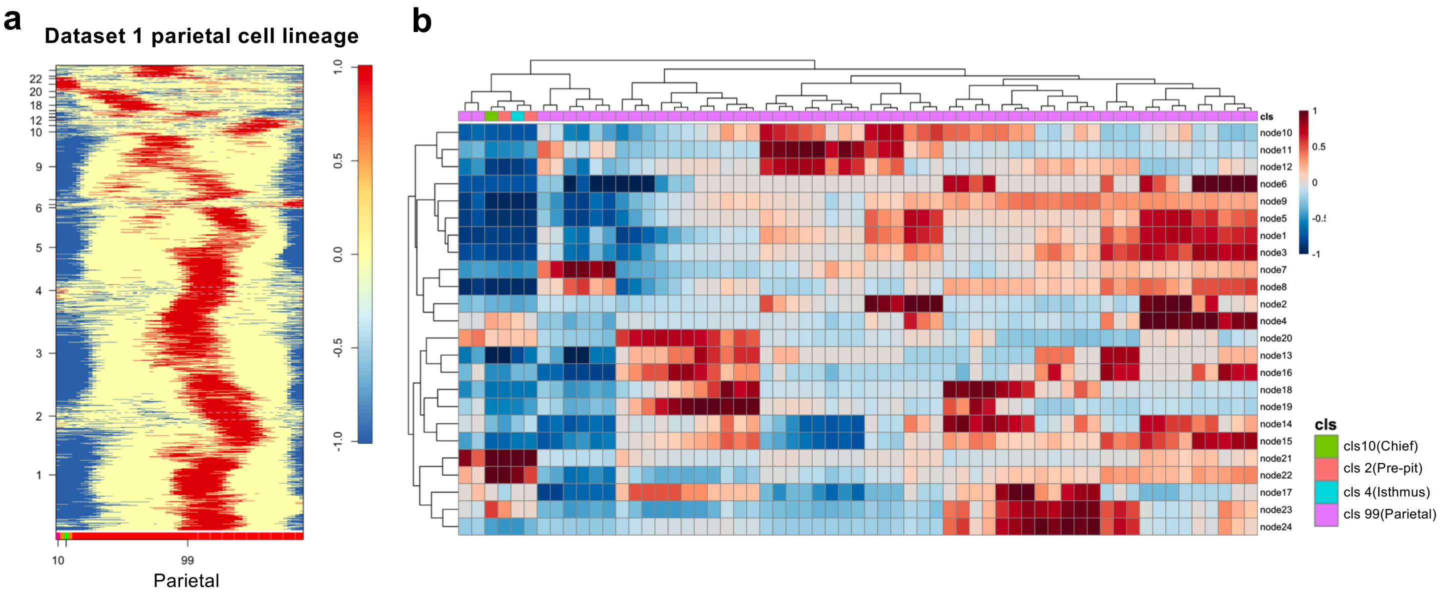

Supplementary Fig. 13. Characterization of the parietal cell differentiation process in dataset 1. Related to Fig.2.

a Self-organizing map of binarized pseudotemporal expression profiles along the parietal cell differentiation trajectory. The x-axis indicates the cells involved in the parietal cell lineage that are colored according to cell types and the y-axis indicates the nodes.

b Hierarchical clustering of co-expression nodes in (a). The x-axis indicates the cells involved in the parietal cell lineage that are colored according to cell types and the y-axis indicates the nodes. The x-axis in (b) is not same as that of (a) because of the new hierarchical clustering of the cells. The colors for each cell type are shown on the right bottom corner.

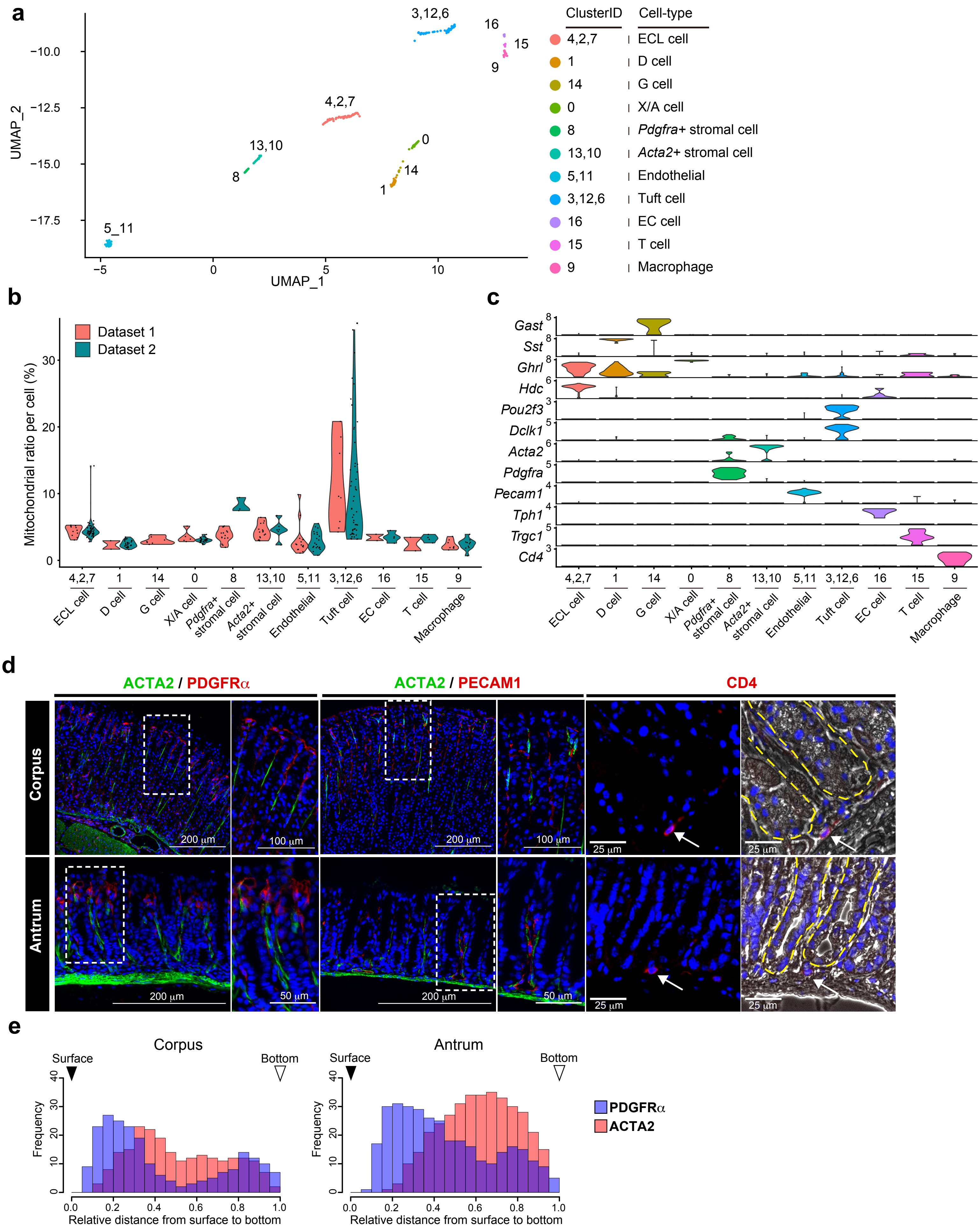
a
b
c
d
e
UMAP_1
UMAP_2
ClusterID
Cell-type
4,2,7 ECL cell
1 D cell
14 G cell
0 X/A cell
8 Pdgfra+ stromal cell
13,10 Acta2+ stromal cell
5,11 Endothelial
3,12,6 Tuft cell
16 EC cell
15 T cell
9 Macrophage
Dataset 1
Dataset 2
Mitochondrial ratio per cell (%)
Gast
Sst
Ghrl
Hdc
Pou2f3
Dclk1
Acta2
Pdgfra
Pecam1
Tph1
Trgc1
Cd4
ACTA2 / PDGFRα
ACTA2 / PECAM1
CD4
Corpus
Antrum
200 μm
100 μm
50 μm
25 μm
Surface
Bottom
Frequency
Relative distance from surface to bottom
PDGFRα
ACTA2

Supplementary Fig. 14. Characterization of gastric cells other than the major corpus epithelial cells. Related to Fig.3.

a UMAP visualization of gastric cells other than the major corpus epithelial identified in datasets 1 and 2.

b Violin plots showing mitochondrial ratio per cell in each cluster. The cells derived from datasets 1 and 2 are in red and green, respectively.

c Violin plots showing the expression of known marker genes in each cluster.

d Immunofluorescence staining of adult mouse corpus and antrum tissues with ACTA2 (green), PDGFRα (red), PECAM1 (red), CD4 (red), and DAPI (blue). High magnification images of the dotted squares are shown on the right side. As for CD4 staining, phase contrast pictures merged with CD4 and DAPI are shown on the right side. Yellow lines indicate each gastric gland. The images are representative of three independent experiments (ACTA2/PDGFRa) or two independent experiments (ACTA2/PECAM1 and CD4).

e Histogram showing the distribution of PDGFRα^{+} (blue) and ACTA2^{+} (red) stromal cells in adult mouse corpus and antrum gastric units. The x-axis indicates the relative position in gastric glands, and the y-axis indicates the number of the stromal cells detected at each position.

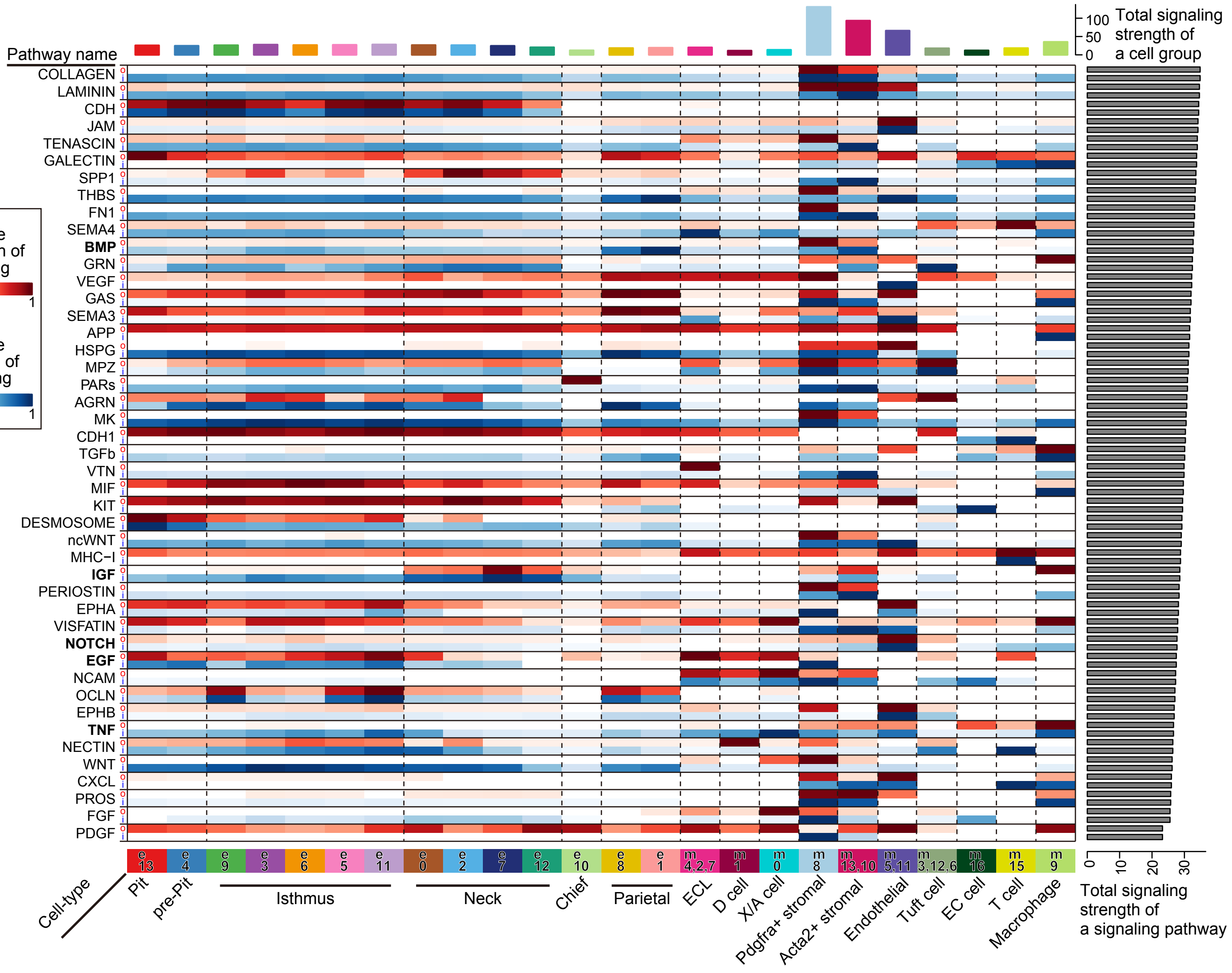

Supplementary Fig. 15. CellChat-based Analysis of outgoing and incoming signaling in each cell type. Related to Fig. 3

The x-axis indicates cell types and the y-axis indicates signaling pathways. For each signaling, the outgoing signal intensity is shown in red (upper row) and the incoming signal intensity is shown in blue (lower row).

a

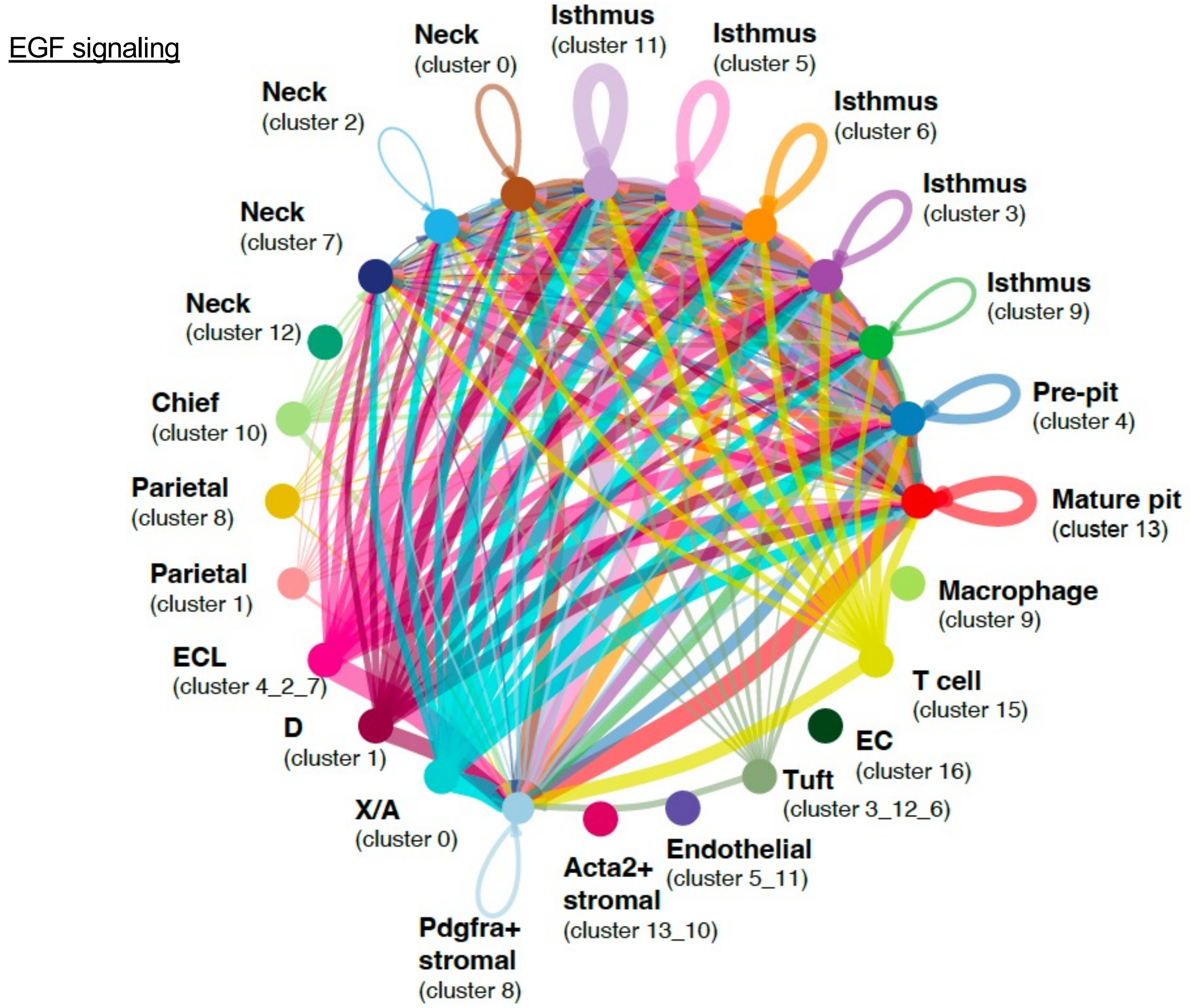
EGF signaling
Neck
(cluster 0)
Isthmus
(cluster 11)
Isthmus
(cluster 5)
Neck
(cluster 2)
Isthmus
(cluster 6)
Neck
(cluster 7)
Isthmus
(cluster 3)
Neck
(cluster 12)
Isthmus
(cluster 9)
Chief
(cluster 10)
Pre-pit
(cluster 4)
Parietal
(cluster 8)
Mature pit
(cluster 13)
Parietal
(cluster 1)
Macrophage
(cluster 9)
ECL
(cluster 4_2_7)
T cell
(cluster 15)
D
(cluster 1)
EC
(cluster 16)
Tuft
(cluster 3_12_6)
X/A
(cluster 0)
Endothelial
(cluster 5_11)
Acta2+
stromal
(cluster 13_10)
Pdgfra+
stromal
(cluster 8)

b

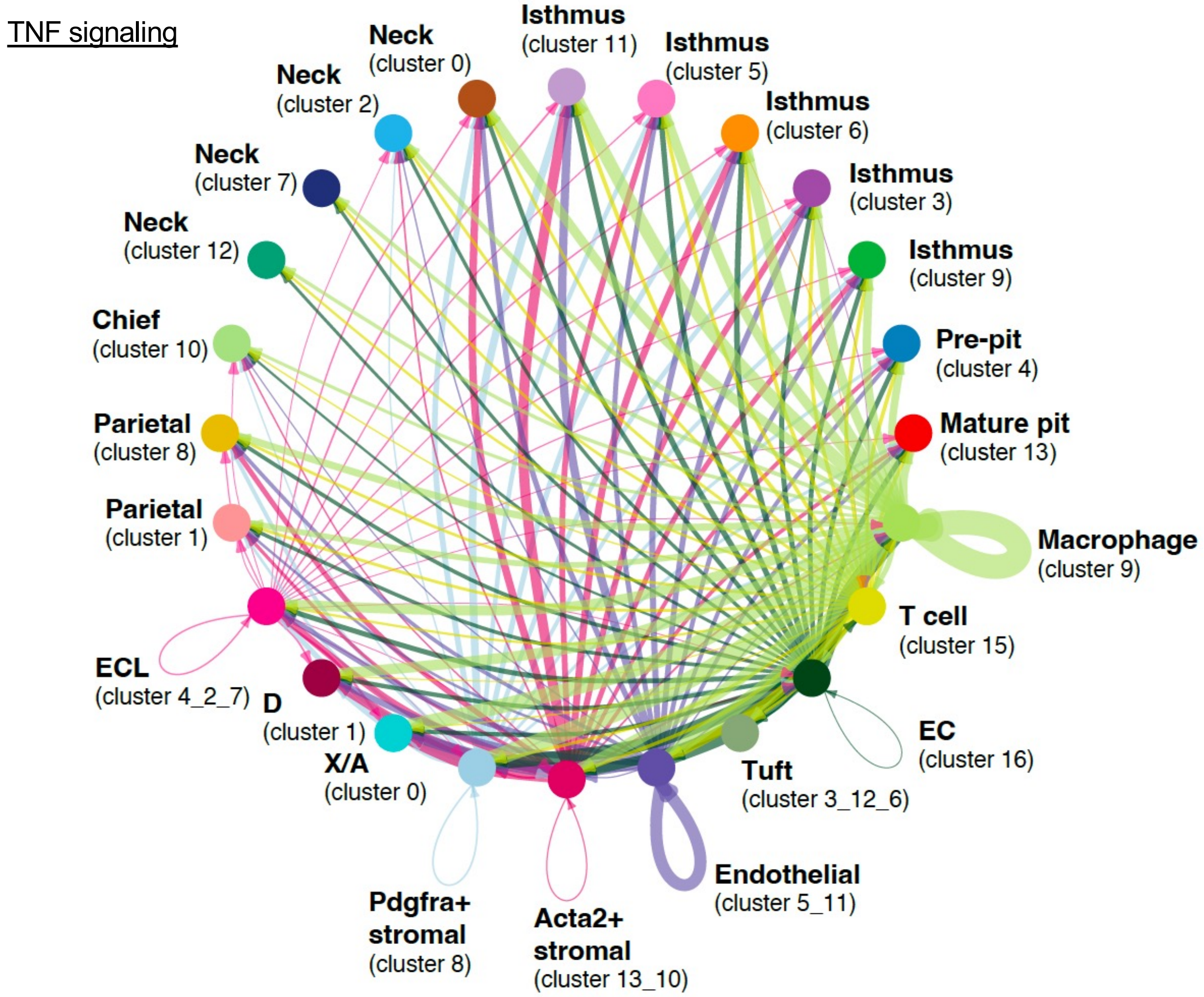
TNF signaling
Neck
(cluster 0)
Isthmus
(cluster 11)
Isthmus
(cluster 5)
Neck
(cluster 2)
Isthmus
(cluster 6)
Neck
(cluster 7)
Isthmus
(cluster 3)
Neck
(cluster 12)
Isthmus
(cluster 9)
Chief
(cluster 10)
Pre-pit
(cluster 4)
Parietal
(cluster 8)
Mature pit
(cluster 13)
Parietal
(cluster 1)
Macrophage
(cluster 9)
T cell
(cluster 15)
ECL
(cluster 4_2_7)
D
(cluster 1)
EC
(cluster 16)
X/A
(cluster 0)
Tuft
(cluster 3_12_6)
Endothelial
(cluster 5_11)
Pdgfra+
stromal
(cluster 8)
Acta2+
stromal
(cluster 13_10)

Supplementary Fig. 16. Signaling networks inferred by CellChat analysis. Related to Fig.3.

CellChat analysis predicted cell-cell communication among all gastric cell types for EGF (a), and TNF (b) signaling pathways. Line width represents communication probabilities.

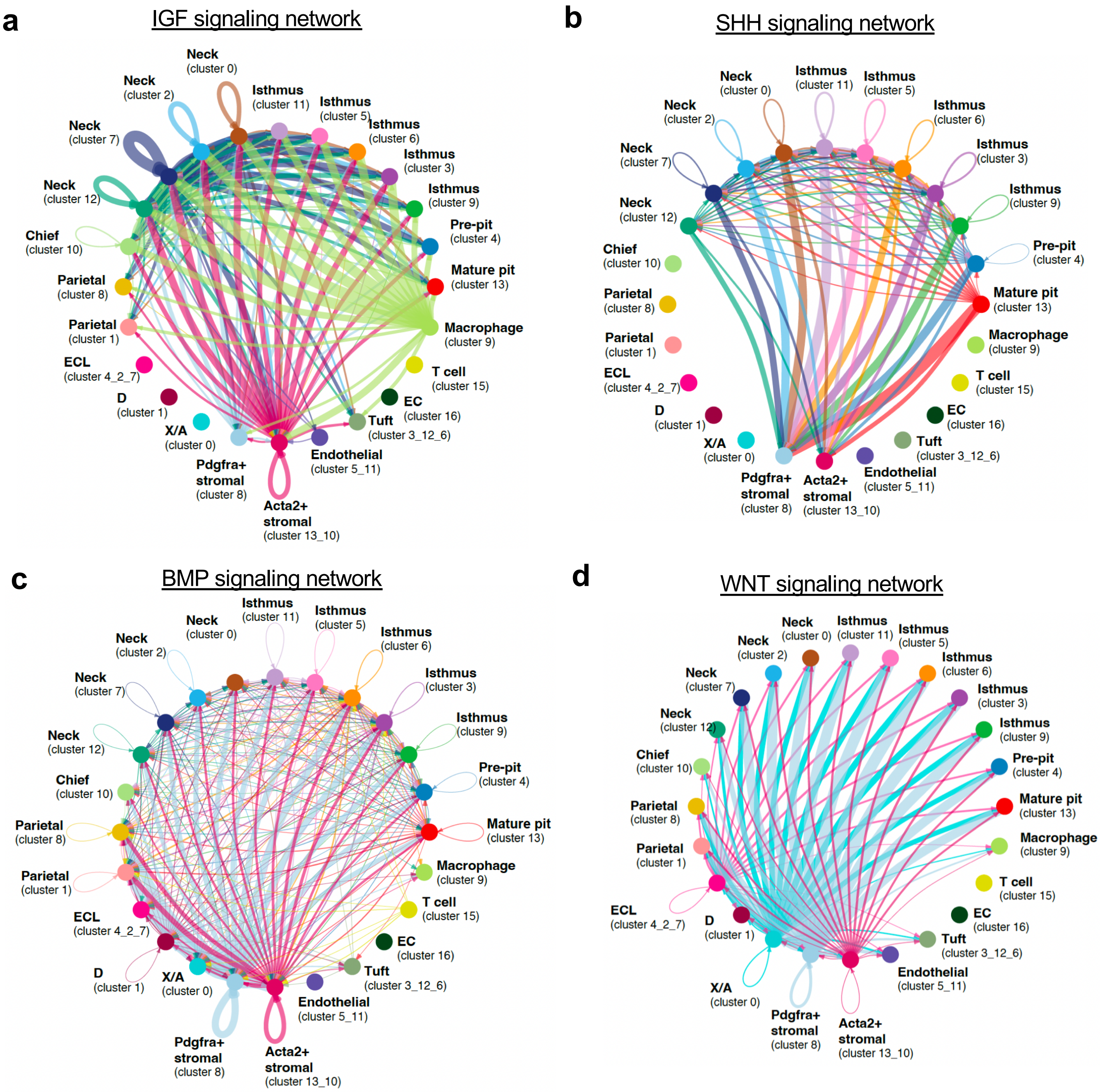
a
IGF signaling network
Neck (cluster 0)
Neck (cluster 2)
Neck (cluster 7)
Neck (cluster 12)
Isthmus (cluster 11)
Isthmus (cluster 5)
Isthmus (cluster 6)
Isthmus (cluster 3)
Isthmus (cluster 9)
Pre-pit (cluster 4)
Mature pit (cluster 13)
Macrophage (cluster 9)
T cell (cluster 15)
EC (cluster 16)
Tuft (cluster 3_12_6)
Endothelial (cluster 5_11)
Acta2+ stromal (cluster 13_10)
Pdgfra+ stromal (cluster 8)
X/A (cluster 0)
D (cluster 1)
ECL (cluster 4_2_7)
Parietal (cluster 1)
Parietal (cluster 8)
Chief (cluster 10)
b
SHH signaling network
Neck (cluster 0)
Isthmus (cluster 11)
Isthmus (cluster 5)
Isthmus (cluster 6)
Isthmus (cluster 3)
Isthmus (cluster 9)
Pre-pit (cluster 4)
Mature pit (cluster 13)
Macrophage (cluster 9)
T cell (cluster 15)
EC (cluster 16)
Tuft (cluster 3_12_6)
Endothelial (cluster 5_11)
Acta2+ stromal (cluster 13_10)
Pdgfra+ stromal (cluster 8)
X/A (cluster 0)
D (cluster 1)
ECL (cluster 4_2_7)
Parietal (cluster 1)
Parietal (cluster 8)
Chief (cluster 10)
Neck (cluster 12)
Neck (cluster 7)
Neck (cluster 2)
c
BMP signaling network
Isthmus (cluster 11)
Isthmus (cluster 5)
Isthmus (cluster 6)
Isthmus (cluster 3)
Isthmus (cluster 9)
Pre-pit (cluster 4)
Mature pit (cluster 13)
Macrophage (cluster 9)
T cell (cluster 15)
EC (cluster 16)
Tuft (cluster 3_12_6)
Endothelial (cluster 5_11)
Acta2+ stromal (cluster 13_10)
Pdgfra+ stromal (cluster 8)
X/A (cluster 0)
D (cluster 1)
ECL (cluster 4_2_7)
Parietal (cluster 1)
Parietal (cluster 8)
Chief (cluster 10)
Neck (cluster 12)
Neck (cluster 7)
Neck (cluster 2)
Neck (cluster 0)
d
WNT signaling network
Neck (cluster 0)
Isthmus (cluster 11)
Isthmus (cluster 5)
Isthmus (cluster 6)
Isthmus (cluster 3)
Isthmus (cluster 9)
Pre-pit (cluster 4)
Mature pit (cluster 13)
Macrophage (cluster 9)
T cell (cluster 15)
EC (cluster 16)
Tuft (cluster 3_12_6)
Endothelial (cluster 5_11)
Acta2+ stromal (cluster 13_10)
Pdgfra+ stromal (cluster 8)
X/A (cluster 0)
D (cluster 1)
ECL (cluster 4_2_7)
Parietal (cluster 1)
Parietal (cluster 8)
Chief (cluster 10)
Neck (cluster 12)
Neck (cluster 7)
Neck (cluster 2)

Supplementary Fig. 17. Signaling networks inferred by CellChat analysis. Related to Fig.3.

CellChat analysis predicted cell-cell communication among all gastric cell types for IGF (a), SHH (b), BMP (c), and WNT (d) signaling pathways. Line width represents communication probabilities.

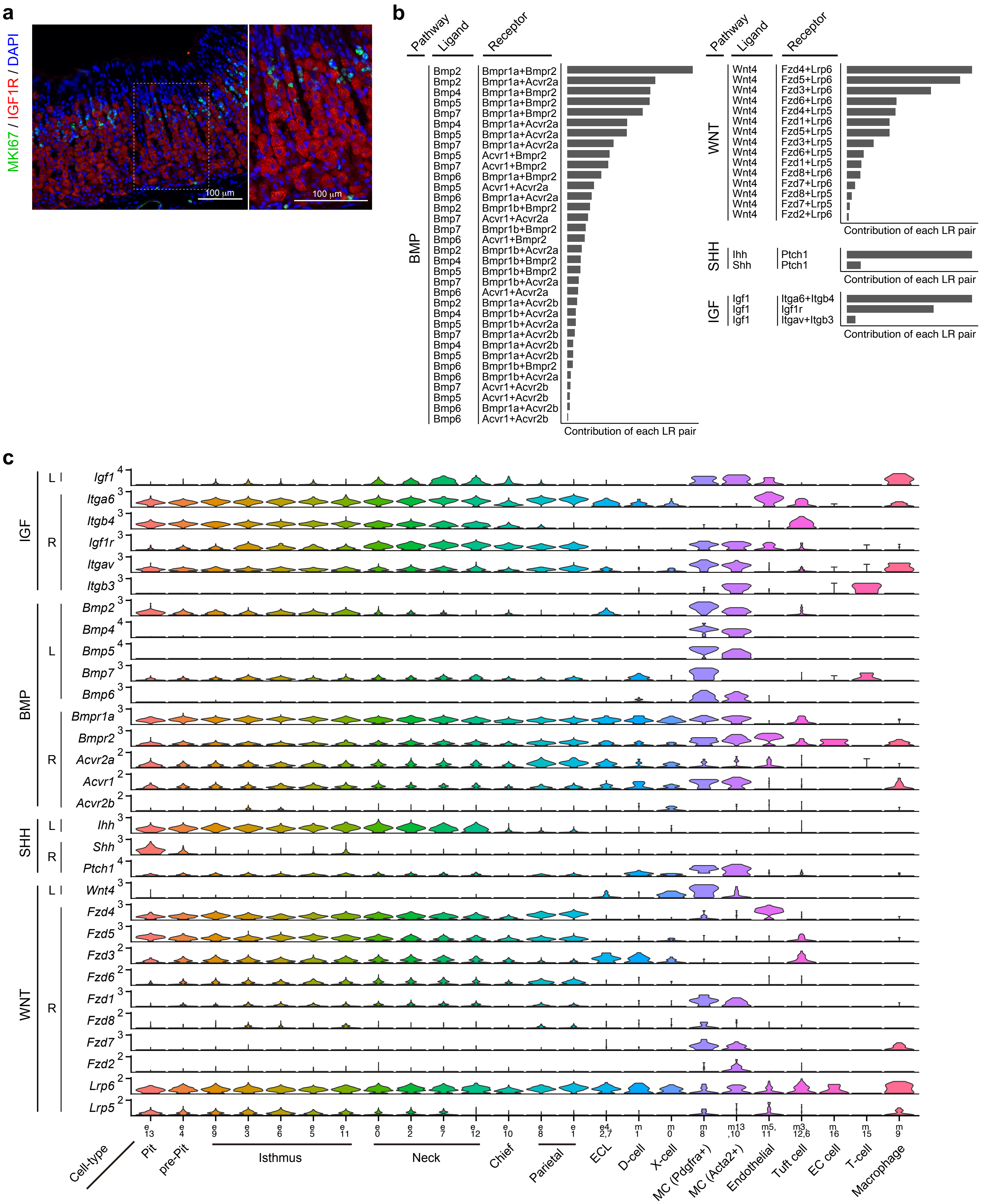
a
MKI67 / IGF1R / DAPI
100 μm
100 μm
b
Pathway
Ligand
Receptor
BMP
Bmp2 Bmpr1a+Bmpr2
Bmp2 Bmpr1a+Acvr2a
Bmp4 Bmpr1a+Bmpr2
Bmp5 Bmpr1a+Bmpr2
Bmp7 Bmpr1a+Bmpr2
Bmp4 Bmpr1a+Acvr2a
Bmp5 Bmpr1a+Acvr2a
Bmp7 Bmpr1a+Acvr2a
Bmp5 Acvr1+Bmpr2
Bmp7 Acvr1+Bmpr2
Bmp6 Bmpr1a+Bmpr2
Bmp5 Acvr1+Acvr2a
Bmp6 Bmpr1a+Acvr2a
Bmp2 Bmpr1b+Bmpr2
Bmp7 Acvr1+Acvr2a
Bmp7 Bmpr1b+Bmpr2
Bmp6 Acvr1+Bmpr2
Bmp2 Bmpr1b+Acvr2a
Bmp4 Bmpr1b+Bmpr2
Bmp5 Bmpr1b+Bmpr2
Bmp7 Bmpr1b+Acvr2a
Bmp6 Acvr1+Acvr2a
Bmp2 Bmpr1a+Acvr2b
Bmp4 Bmpr1b+Acvr2a
Bmp5 Bmpr1b+Acvr2a
Bmp7 Bmpr1a+Acvr2b
Bmp4 Bmpr1a+Acvr2b
Bmp5 Bmpr1a+Acvr2b
Bmp6 Bmpr1b+Bmpr2
Bmp6 Bmpr1b+Acvr2a
Bmp7 Acvr1+Acvr2b
Bmp5 Acvr1+Acvr2b
Bmp6 Bmpr1a+Acvr2b
Bmp6 Acvr1+Acvr2b
Contribution of each LR pair
WNT
Wnt4 Fzd4+Lrp6
Wnt4 Fzd5+Lrp6
Wnt4 Fzd3+Lrp6
Wnt4 Fzd6+Lrp6
Wnt4 Fzd4+Lrp5
Wnt4 Fzd1+Lrp6
Wnt4 Fzd5+Lrp5
Wnt4 Fzd3+Lrp5
Wnt4 Fzd6+Lrp5
Wnt4 Fzd1+Lrp5
Wnt4 Fzd8+Lrp6
Wnt4 Fzd7+Lrp6
Wnt4 Fzd8+Lrp5
Wnt4 Fzd7+Lrp5
Wnt4 Fzd2+Lrp6
Contribution of each LR pair
SHH
Ihh Ptch1
Shh Ptch1
Contribution of each LR pair
IGF
Igf1 Itga6+Itgb4
Igf1 Igf1r
Igf1 Itgav+Itgb3
Contribution of each LR pair
c
IGF L Igf1 4
R Itga6 3
Itgb4 3
Igf1r 3
Itgav 3
Itgb3 3
BMP L Bmp2 3
Bmp4 4
Bmp5 4
Bmp7 3
Bmp6 3
R Bmpr1a 3
Bmpr2 3
Acvr2a 2
Acvr1 2
Acvr2b 2
SHH L Ihh 3
R Shh 3
Ptch1 4
WNT L Wnt4 3
R Fzd4 3
Fzd5 3
Fzd3 2
Fzd6 2
Fzd1 3
Fzd8 2
Fzd7 3
Fzd2 2
Lrp6 2
Lrp5 2
e13 e4 e9 e3 e6 e5 e11 e0 e2 e7 e12 e10 e8 e1 e4,2,7 m1 m0 m8 m13,10 m5,11 m3,12,6 m16 m15 m9
Cell-type
Pit
pre-Pit
Isthmus
Neck
Chief
Parietal
ECL
D-cell
X-cell
MC (Pdgfra+)
MC (Acta2+)
Endothelial
Tuft cell
EC cell
T-cell
Macrophage

Supplementary Fig. 18. Expression pattern of major inferred ligand-receptor pairs for each signaling pathway.

a Immunofluorescence staining of MKI67 (green), IGF1R (red), and DAPI (blue) in adult mouse corpus tissues. High magnification images of the dotted squares are shown on the right side. The images are representative of two independent experiments.

b Relative contribution of BMP, WNT, SHH, and IGF ligand-receptor pairs calculated by CellChat.

c Violin plots showing the expression of IGF, BMP, SHH, and WNT ligands and receptors that were identified as major contributors by CellChat.

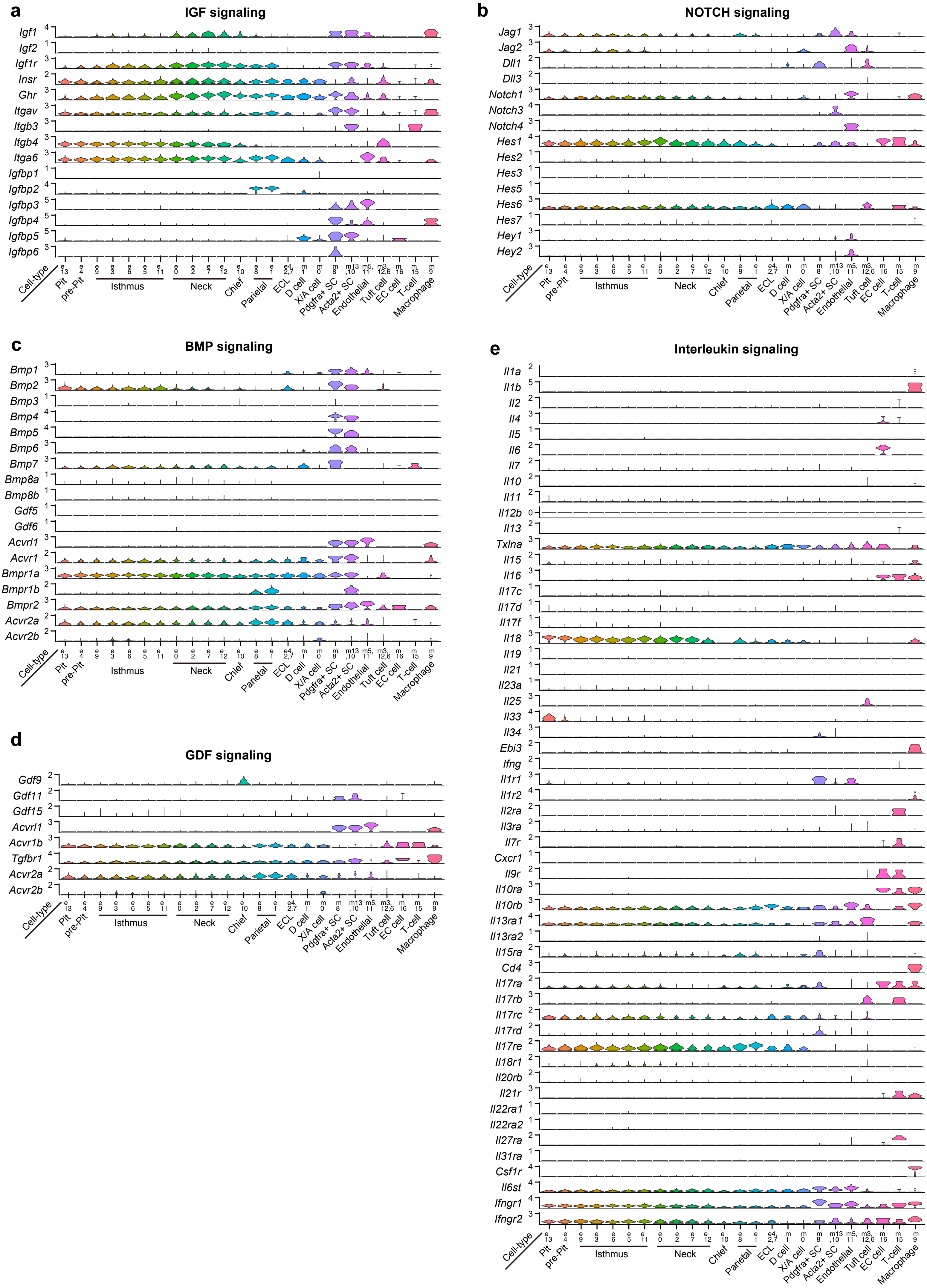
a
IGF signaling
Igf1
Igf2
Igf1r
Insr
Ghr
Itgav
Itgb3
Itgb4
Itga6
Igfbp1
Igfbp2
Igfbp3
Igfbp4
Igfbp5
Igfbp6
b
NOTCH signaling
Jag1
Jag2
Dll1
Dll3
Notch1
Notch3
Notch4
Hes1
Hes2
Hes3
Hes5
Hes6
Hes7
Hey1
Hey2
c
BMP signaling
Bmp1
Bmp2
Bmp3
Bmp4
Bmp5
Bmp6
Bmp7
Bmp8a
Bmp8b
Gdf5
Gdf6
Acvrl1
Acvr1
Bmpr1a
Bmpr1b
Bmpr2
Acvr2a
Acvr2b
d
GDF signaling
Gdf9
Gdf11
Gdf15
Acvrl1
Acvr1b
Tgfbr1
Acvr2a
Acvr2b
e
Interleukin signaling
Il1a
Il1b
Il2
Il4
Il5
Il6
Il7
Il10
Il11
Il12b
Il13
Txlna
Il15
Il16
Il17c
Il17d
Il17f
Il18
Il19
Il21
Il23a
Il25
Il33
Il34
Ebi3
Ifng
Il1r1
Il1r2
Il2ra
Il3ra
Il7r
Cxcr1
Il9r
Il10ra
Il10rb
Il13ra1
Il13ra2
Il15ra
Cd4
Il17ra
Il17rb
Il17rc
Il17rd
Il17re
Il18r1
Il20rb
Il21r
Il22ra1
Il22ra2
Il27ra
Il31ra
Csf1r
Il6st
Ifngr1
Ifngr2
Cell-type
e13 e4 e9 e3 e6 e5 e11 e0 e2 e7 e12 e10 e8 e1 e4,2,7 m1 m0 m8 m13,10 m5,11 m3,12,6 m16 m15 m9
Pit
pre-Pit
Isthmus
Neck
Chief
Parietal
ECL
D cell
X/A cell
Pdgfra+ SC
Acta2+ SC
Endothelial
Tuft cell
EC cell
T-cell
Macrophage

Supplementary Fig. 19. Expression pattern of ligands, receptors, and downstream regulators for IGF (a), NOTCH (b), BMP (c), GDF (d), and Interleukin (e) signaling pathways across all gastric cell types.

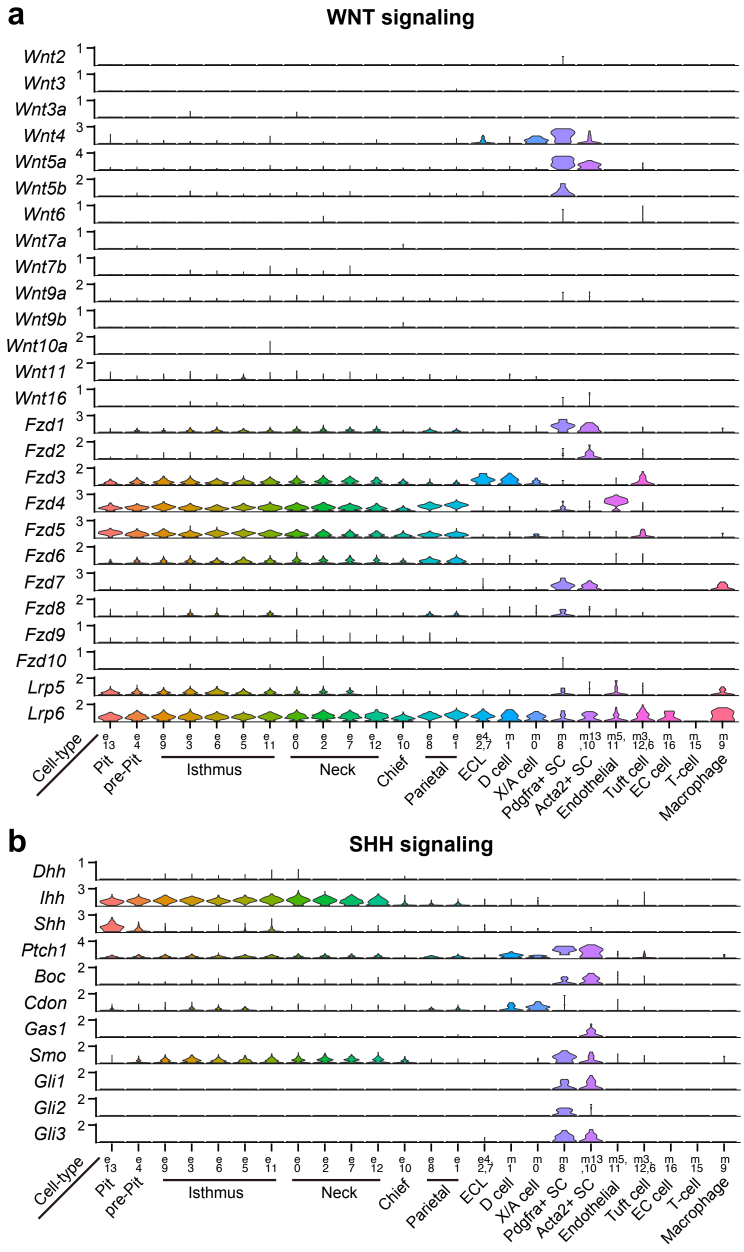

Supplementary Fig. 20. Expression pattern of ligands and receptors for WNT (a) and SHH (b) signaling pathway across all gastric cell types.

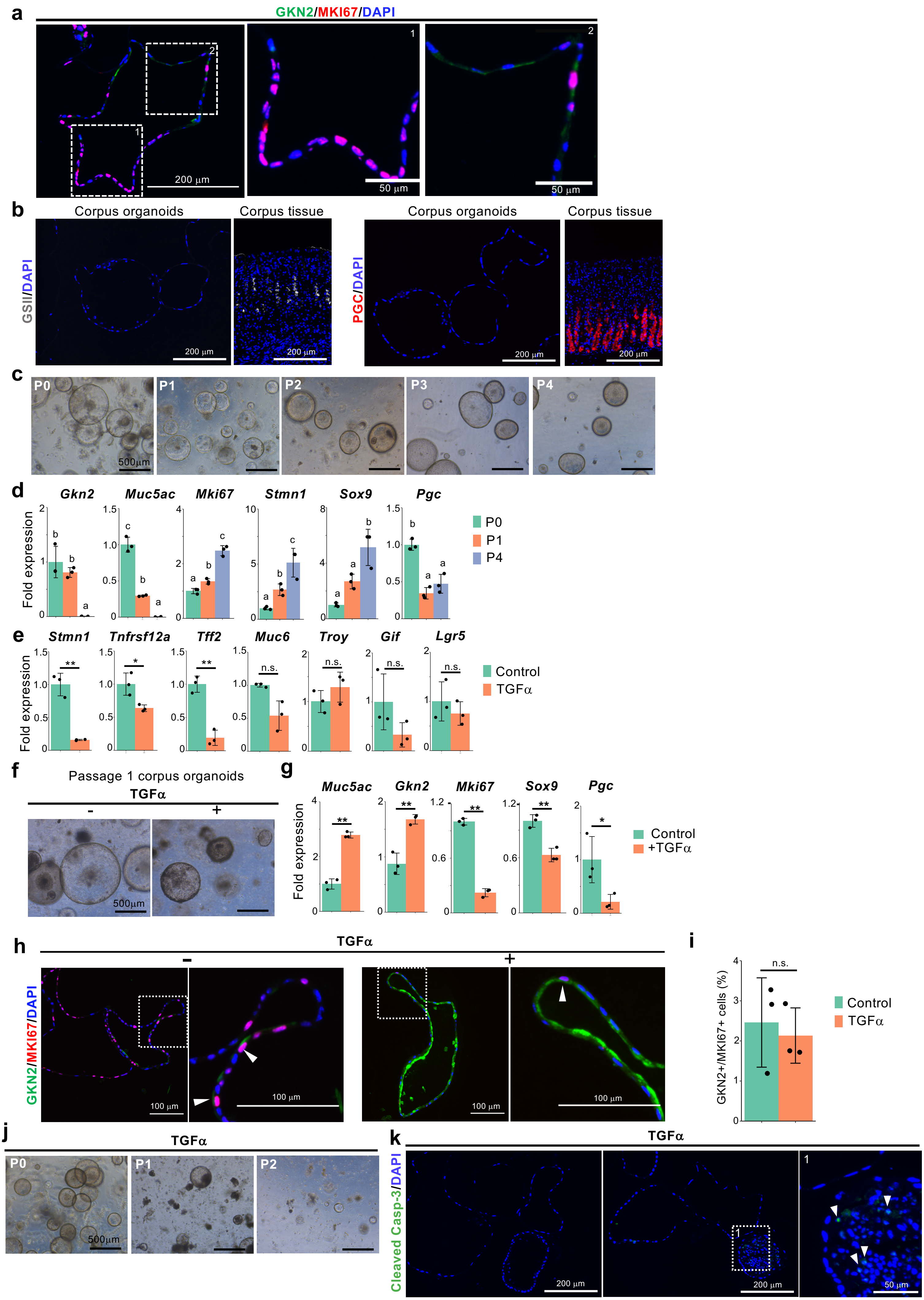

a
GKN2/MKI67/DAPI
200 µm
50 µm
b
Corpus organoids
Corpus tissue
GSII/DAPI
PGC/DAPI
200 µm
c
P0
P1
P2
P3
P4
500µm
d
Gkn2
Muc5ac
Mki67
Stmn1
Sox9
Pgc
Fold expression
P0
P1
P4
e
Stmn1
Tnfrsf12a
Tff2
Muc6
Troy
Gif
Lgr5
Control
TGFα
f
Passage 1 corpus organoids
TGFα
g
Muc5ac
Gkn2
Mki67
Sox9
Pgc
Control
+TGFα
h
TGFα
GKN2/MKI67/DAPI
100 µm
i
GKN2+/MKI67+ cells (%)
n.s.
j
TGFα
k
TGFα
Cleaved Casp-3/DAPI

Supplementary Fig. 21. TGFα promotes pit cell differentiation and suppresses cell proliferation. Related to Fig. 4.

a Immunofluorescence staining of GKN2 (green), MKI67 (red), and DAPI (blue) in corpus organoids prepared from adult mouse corpus. $MKI67^{-}$ cells frequently express low level of GKN2. High magnification images of the dotted squares are shown on the right side.

b Immunofluorescence staining of GSII (white), PGC (red), and DAPI (blue) in corpus organoids and adult corpus tissue. The images are representative of two independent experiments.

c Corpus organoids cultured in the control medium at passage 0–4. Scale bar, 500 µm.

d qPCR analysis of gastric epithelial cell marker expression in the corpus organoids at passage 0, 1, and 4 in (c). Data are presented as mean fold changes ± SD (*n* = 3 biologically independent samples). P1 vs P0 *n.s.*, P4 vs P0 *p* = 0,0010, P4 vs P1 *p* = 0.0032 for *Gkn2*; *p* < 0.0001, *p* < 0.0001, *p* = 0.0025 for *Muc5ac*; *p* = 0.0400, *p* < 0.0001, *p* = 0.0001 for *Mki67*; *p* = 0.0334, *p* < 0.0001, *p* = 0.0003 for *Stmn1*; *n.s.*, *p* = 0.0020, *p* = 0.0240 for *Sox9*; *p* = 0.0004, *p* = 0.0013, *n.s.* for *Pgc*.

e qPCR analysis of gastric epithelial cell marker expression in the organoids in Fig. 4a. Data are presented as mean fold changes ± SD (n = 3 biologically independent samples for each culture condition). ***p* = 0.0011 for *Stmn1*; **p* = 0.0230 for *Tnfrsf12a*; ***p* = 0.0011 for *Tff2*.

f Passage 1 corpus organoids treated with or without TGFα. The organoids were grown without TGFα for 6 days and then dissociated with a recombinant enzyme, TrypLE Express, to generate passage 1 organoids. The passage 1 organoids were further grown with or without TGFα (25 ng/mL) for 6 days. Scale bar, 500 µm.

g qPCR analysis of gastric epithelial marker expression in corpus organoids in (f). Data are presented as mean fold changes ± SD (n = 3 biologically independent samples for each culture condition). ***p* = 0.0001 for *Muc5ac*; ***p* = 0.0027 for *Gkn2*; ***p* < 0.0001 for *Mki67*; ***p* = 0.0029 for *Sox9*; **p* = 0.0397 for *Pgc*.

h Double immunofluorescence staining of GKN2 (green) and MKI67 (red) in corpus organoids treated with or without TGFα (12.5 ng/mL) for 6 days. Arrowheads indicate $GKN2^{+}/MKI67^{+}$ cells. Insets show high magnification images of boxed areas.

i Quantification of $GKN2^{+}/MKI67^{+}$ cells in corpus organoids in (h). Data are presented as mean fold changes ± SD (*n* = 3 biologically independent samples for each culture condition).

j Gastric organoids treated with TGFα (12.5 ng/mL) from the beginning of the culture at passage 0, 1, and 2. The number of organoids was dramatically decreased after first passage. Scale bar, 500 µm. The images are representative of two independent experiments.

k Immunofluorescence staining of cleaved caspase-3 (green) with nuclear DAPI staining (blue) in corpus organoids treated with TGFα (12.5 ng/mL) for 6 days. Arrowheads indicate cleaved caspase-3^{+} shed cells in the lumen. High magnification images of the dotted squares are shown on the right side. The images are representative of two independent experiments.

Statistical information: significance was calculated by two-tailed Student's *t*-tests for samples with equal variances or two-sided Welch's *t*-tests for samples with unequal variances in (e, g, and i) (**p* < 0.05; ***p* < 0.01); significance was calculated by one-way ANOVA followed by Tukey post hoc test at the 0.05 significance level in (d). Source data are provided as a Source Data file.

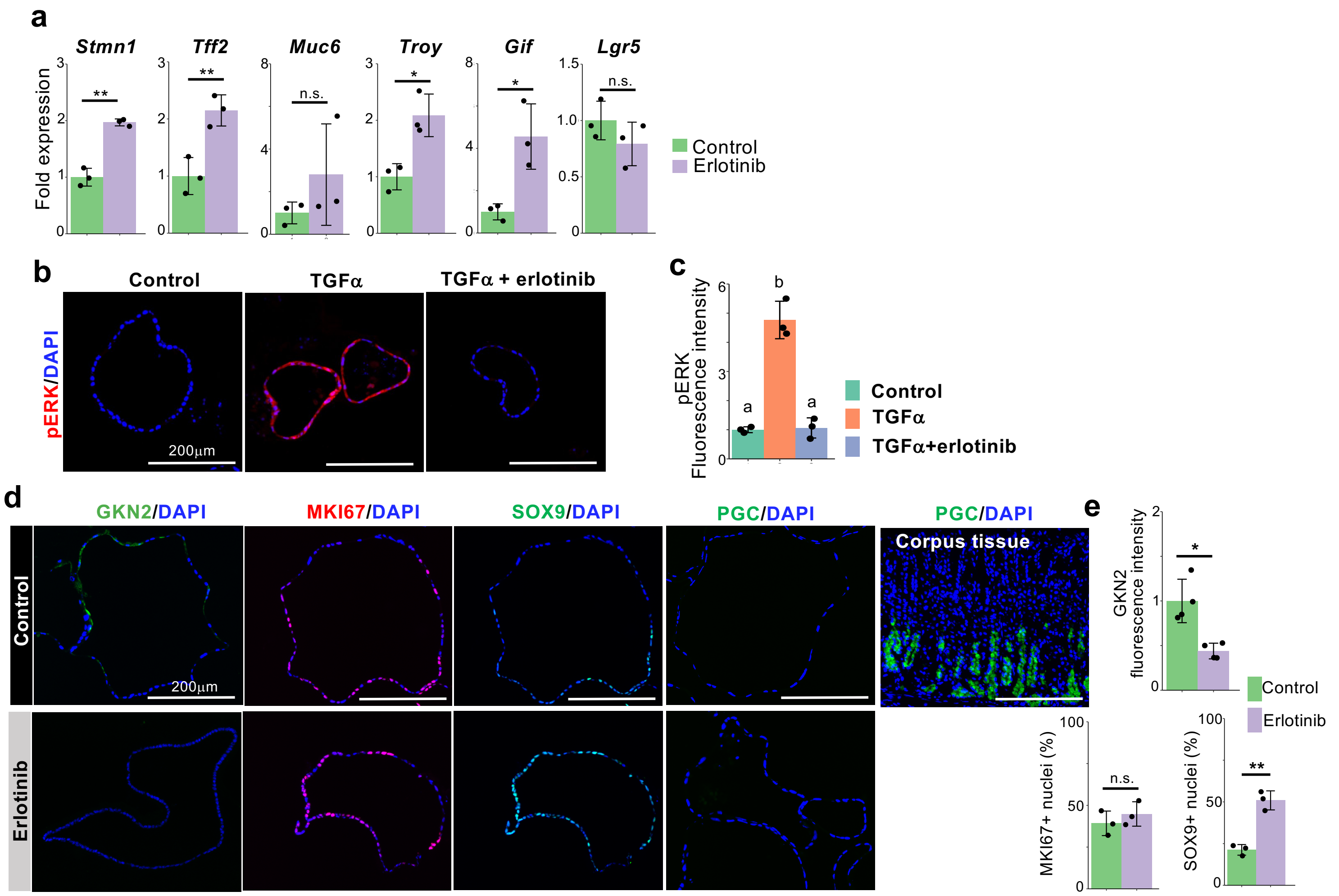

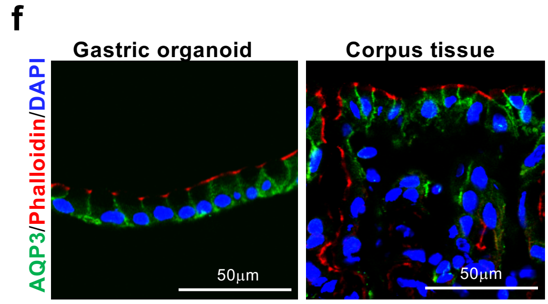

g

Barker et al. (2010)	Our study
Wnt3a-conditioned medium<br>R-spondin1: 1 µg/mL<br>Noggin: 100 ng/mL<br>FGF10: 100 ng/mL<br>Gastrin: 10 nM<br>EGF: 50 ng/mL	Wnt3a (recombinant): 100 ng/mL<br>R-spondin1: 1 µg/mL<br>LDN: 0.1 µM<br>FGF10: 100 ng/mL<br>Gastrin: 10 nM<br>Y27632: 10 µM

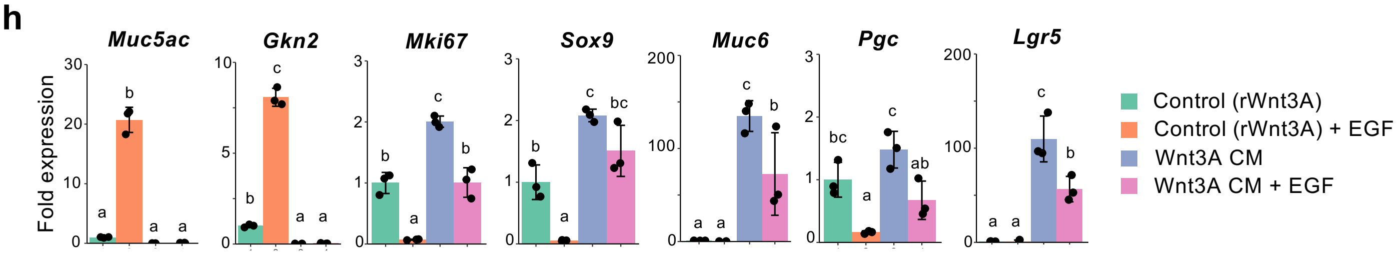

Supplementary Fig. 22. Inhibition of EGFR signaling suppresses pit cell differentiation. Related to Fig. 4.

a qPCR analysis of gastric epithelial cell marker expression in erlotinib-treated corpus organoids in Fig. 4f. Data are presented as mean fold changes ± SD (*n* = 3 biologically independent samples for each culture condition). ***p* = 0.0005 for *Stmn1*; ***p* = 0.0093 for Tff2; **p* = 0.0131 for *Troy*; **p* = 0.0179 for *Gif*.

b Immunofluorescence analysis of pERK (red) and DAPI (blue) in corpus organoids cultured with or without TGFα and erlotinib. Scale bar, 200 μm.

c Quantification of pERK fluorescence intensity in corpus organoids in (b). Data are presented as mean fold changes ± SD (*n* = 3 biologically independent samples). Each data point represents the mean value of at least ten organoids. TGFα vs Control *p* < 0.0001, TGFα + erlotinib vs Control *n.s.*, TGFα + erlotinib vs TGFα *p* < 0.0001.

d Immunofluorescence staining of GKN2 (green), MKI67 (red), SOX9 (green), PGC (green), and DAPI (blue) in corpus organoids treated with or without erlotinib (0.5 μM). The adult mouse corpus tissues stained with PGC (green) is shown in the upper right corner as a positive control. Scale bar, 200 μm.

e Quantification of GKN2 fluorescence intensity and the percentage of $MKI67^+$ and $SOX9^+$ cells in the corpus organoids in (d). Data are presented as mean fold changes ± SD (*n* = 3 or 4 biologically independent samples). ***p* = 0.0049 for GKN2 fluorescence intensity; ***p* = 0.0014 for $SOX9^+$ nuclei.

f The corpus organoid and the corpus tissue stained with anti-AQP3 antibody (green), phalloidin (red), and DAPI (blue). Images were captured by laser confocal microscopy and representative of three independent experiments.

g Comparison of growth factors and chemicals used in the previous study (Barker et al. 2010) and those used in our study.

h qPCR analysis of gastric epithelial cell marker expression in the corpus organoids cultured with rWnt3a (100 ng/mL) or Wnt3a CM. The response to EGF (50 ng/mL) was analyzed. Data are presented as mean fold changes ± SD (*n* = 3 biologically independent samples for each culture condition). Control + EGF vs Control *p* < 0.001, WNT3A CM vs Control *n.s.*, WNT3A CM + EGF vs Control *n.s.*, WNT3A CM vs Control + EGF *p* < 0.0001, WNT3A CM + EGF vs Control + EGF *p* < 0.0001, WNT3A CM + EGF vs WNT3A CM *n.s.* for *Muc5ac*; *p* < 0.0001, *p* = 0.0060, *p* = 0.0066, *p* < 0.0001, *p* < 0.0001, *n.s.* for *Gkn2*; *p* = 0.0004, *p* = 0.0002, *n.s.*, *p* < 0.0001, *p* = 0.0003, *p* = 0.0002 for *Mki67*; *p* = 0.0082, *p* = 0.0037, n.s., *p* < 0.0001, *p* = 0.0005, *n.s.* for *Sox9*; *n.s.*, *p* = 0.0006, *p* = 0.02486, *p* = 0.0005, *p* = 0.0235, *p* = 0.0486 for *Muc6*; *p* = 0.01548, *n.s.*, *n.s.*, *p* = 0.0010, *n.s.*, *p* = 0.0191 for *Pgc*; *n.s.*, *p* < 0.0001, *p* = 0.0053, *p* < 0.0001, *p* = 0.0053, *p* = 0.0069 for *Lgr5*.

Statistical information: significance was calculated by two-tailed Student's *t*-tests for samples with equal variances or two-sided Welch's *t*-tests for samples with unequal variances in (a) and (e) (**p* < 0.05; ***p* < 0.01); significance was calculated by one-way ANOVA followed by Tukey post hoc test at the 0.05 significance level in (c) and (h). Source data are provided as a Source Data file.

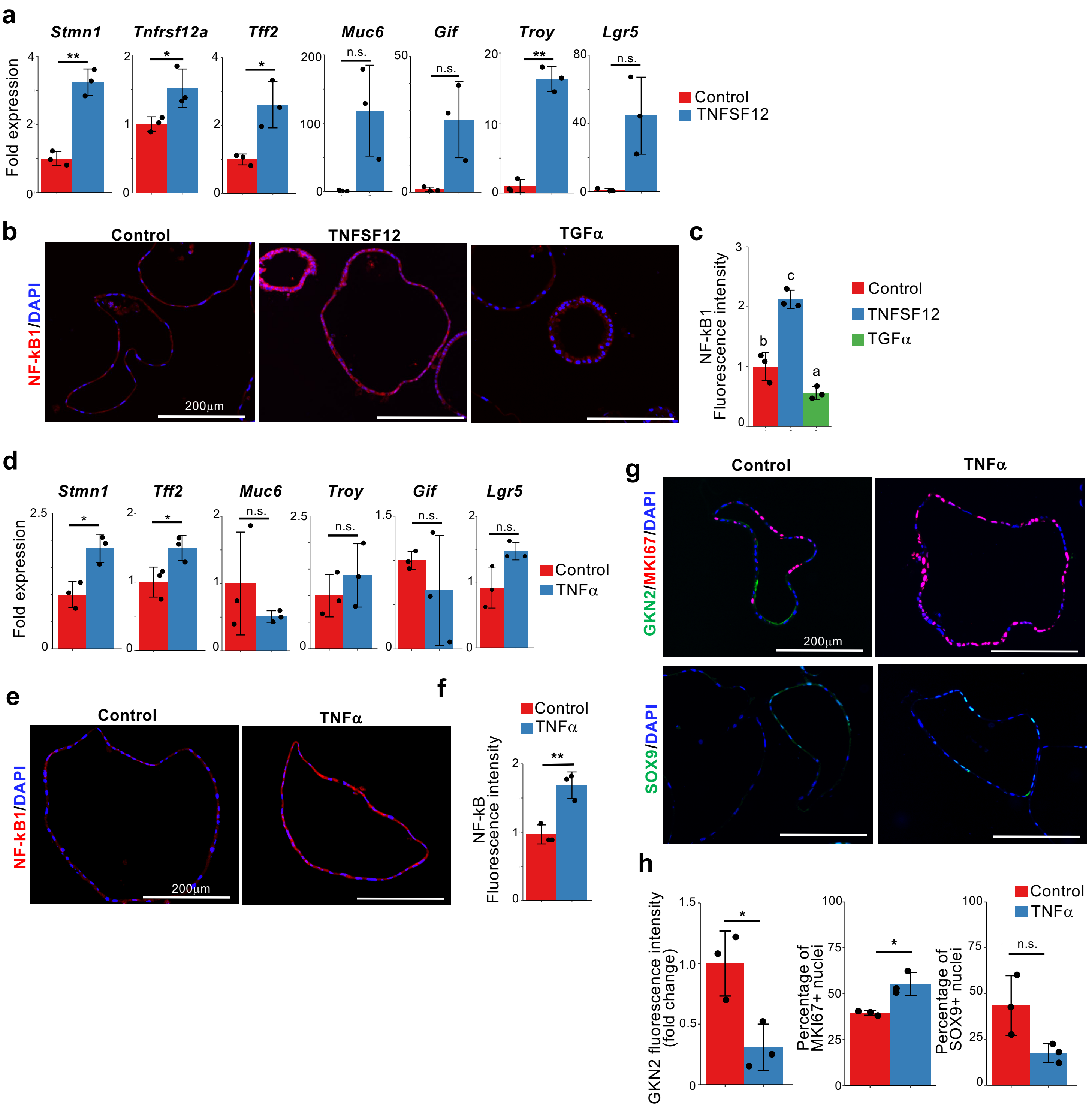

a
Stmn1
Tnfrsf12a
Tff2
Muc6
Gif
Troy
Lgr5
Fold expression
Control
TNFSF12
b
Control
TNFSF12
TGFα
NF-kB1/DAPI
200μm
c
NF-kB1
Fluorescence intensity
Control
TNFSF12
TGFα
d
Stmn1
Tff2
Muc6
Troy
Gif
Lgr5
Fold expression
Control
TNFα
e
Control
TNFα
NF-kB1/DAPI
200μm
f
Control
TNFα
NF-kB
Fluorescence intensity
g
Control
TNFα
GKN2/MKI67/DAPI
200μm
SOX9/DAPI
h
GKN2 fluorescence intensity
(fold change)
Percentage of
MKI67+ nuclei
Percentage of
SOX9+ nuclei
Control
TNFα

Supplementary Fig. 23. Activation of NF-κB signaling suppresses pit cell differentiation. Related to Fig. 5.

a qPCR analysis of gastric epithelial cell markers in the corpus gastric organoids cultured with or without 100 ng/mL TNFSF12 in Fig. 5a. Data are presented as mean fold changes ± SD (*n* = 3 biologically independent samples for each culture condition). **p = 0.0009 for *Stmn1*; *p = 0.0390 for Tnfrsf12a; *p = 0.0164 for *Tff2*; **p = 0.0009 for *Troy*.

b Immunofluorescence analysis of NF-κB1 (red) and DAPI (blue) in the corpus gastric organoids cultured with or without 100 ng/mL TNFSF12 and 12.5 ng/mL TGFα. Scale bar, 200 μm.

c Quantification of the fluorescence intensity of NF-κB1 in the corpus organoids in (b). Data are presented as mean fold changes ± SD (*n* = 3 biologically independent samples). Each data point represents the mean value of at least ten organoids. TGFα vs Control p = 0.0485, TNFSF12 vs Control p = 0.0006, TNFSF12 vs TGFα $p < 0.0001$.

d qPCR analysis of gastric epithelial cell markers in corpus gastric organoids cultured with or without 100 ng/mL TNFα in Fig. 5e. Data are presented as mean fold changes ± SD (*n* = 3 biologically independent samples for each culture condition). *p = 0.0139 for *Stmn1*; *p = 0.0393 for *Tff2*.

e Immunofluorescence staining of NF-κB1 (red) and DAPI (blue) in the corpus gastric organoids cultured with or without 100 ng/mL TNFα. Scale bar, 200 μm.

f Quantification of the fluorescence intensity of NF-κB1 in the gastric organoids in (e). Data are presented as mean fold changes ± SD (*n* = 3 biologically independent samples). Each data point represents the mean value of ten organoids. **p = 0.0066.

g Immunofluorescence staining of GKN2 (green), MKI67 (red), SOX9 (green), and DAPI (blue) in the corpus gastric organoids cultured with or without 100 ng/mL TNFα. Scale bar, 200 μm.

h Quantification of GKN2 fluorescence intensity and the percentage of $MKI67^+$ and $SOX9^+$ cells of the corpus gastric organoids in (g). Data are presented as mean fold changes ± SD (*n* = 3 biologically independent samples). Each data point represents the mean value of at least ten organoids. *p = 0.0220 for GKN2 fluorescence intensity, *p = 0.0126 for $MKI67^+$ nuclei.

Statistical information: significance was calculated by two-tailed Student's *t*-tests for samples with equal variances or two-sided Welch's *t*-tests for samples with unequal variances in (a), (d), (f), and (h)(*$p < 0.05$; ** $p < 0.01$); significance was calculated by one-way ANOVA followed by Tukey post hoc test at the 0.05 significance level in (c). Source data are provided as a Source Data file.

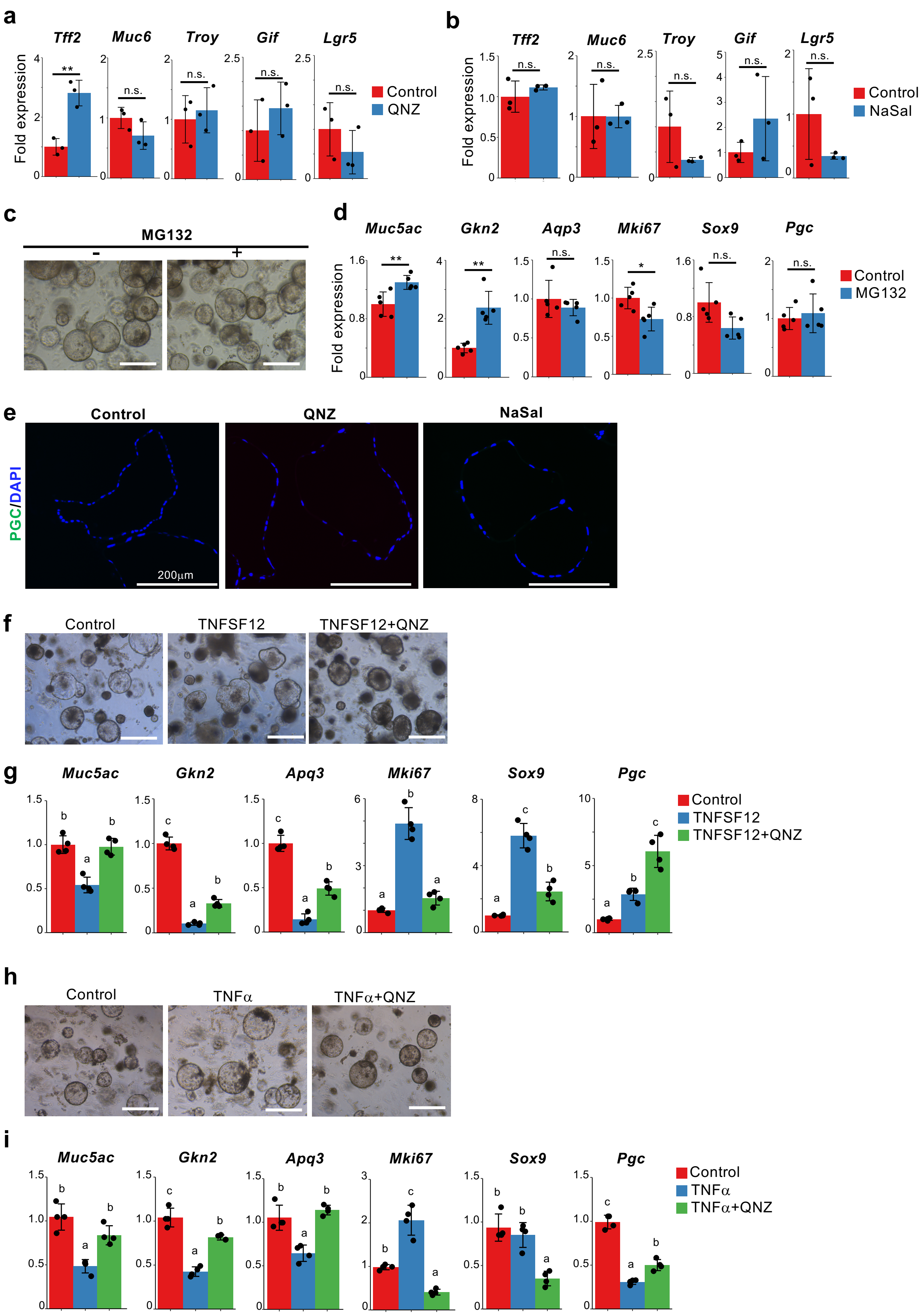

a
Tff2
Muc6
Troy
Gif
Lgr5
Fold expression
**
n.s.
Control
QNZ
b
Tff2
Muc6
Troy
Gif
Lgr5
Fold expression
n.s.
Control
NaSal
c
MG132
-
+
d
Muc5ac
Gkn2
Aqp3
Mki67
Sox9
Pgc
Fold expression
**
*
n.s.
Control
MG132
e
Control
QNZ
NaSal
PGC/DAPI
200µm
f
Control
TNFSF12
TNFSF12+QNZ
g
Muc5ac
Gkn2
Apq3
Mki67
Sox9
Pgc
Control
TNFSF12
TNFSF12+QNZ
h
Control
TNFα
TNFα+QNZ
i
Muc5ac
Gkn2
Apq3
Mki67
Sox9
Pgc
Control
TNFα
TNFα+QNZ

Supplementary Fig. 24. Inhibition of NF-κB signaling induces pit cell differentiation. Related to Fig. 6.

a qPCR analysis of gastric epithelial cell markers in corpus gastric organoids cultured with or without a NF-κB inhibitor, QNZ (16 nM) in Fig. 6a. Data are presented as mean fold changes ± SD (n = 3 biologically independent samples). ** $p = 0.0036$ for *Tff2*.

b qPCR analysis of gastric epithelial cell marker expression in corpus gastric organoids cultured with or without a NF-κB inhibitor NaSal (1.25 mM) in Fig. 6c. Data are presented as mean fold changes ± SD (n = 3 biologically independent samples).

c Corpus organoids cultured with or without a proteosome inhibitor, MG132 (3 μM). Scale bar, 500 μm.

d qPCR analysis of gastric epithelial cell marker expression in the gastric organoids in (c). Data are presented as mean fold changes ± SD (n = 5 biologically independent samples). **$p = 0.0086$ for *Muc5ac*, **$p = 0.0041$ for *Gkn2*, *$p = 0.0189$ for *Mki67*.

e Immunofluorescence staining of PGC (green) and DAPI (blue) in the corpus gastric organoids cultured with or without 16 nM QNZ or 1.25 mM NaSal. Scale bar, 200 μm. The images are representative of two biologically independent samples.

f Corpus organoids cultured with or without TNFSF12 (100 ng/mL) and a NF-κB inhibitor QNZ (80 nM). Scale bar, 500 μm.

g qPCR analysis of gastric epithelial cell marker expression in the corpus organoids in (f). Data are presented as mean fold changes ± SD (n = 4 biologically independent samples). TNFSF12 vs Control $p = 0.0002$, TNFSF12 + QNZ vs Control *n.s.*, TNFSF12 + QNZ vs TNFSF12 $p = 0.0003$ for *Muc5ac*; $p < 0.0001$, $p < 0.0001$, $p = 0.0003$ for *Gkn2*; $p < 0.0001$, $p < 0.0001$, $p = 0.0003$ for *Aqp3*; $p < 0.0001$, *n.s.*, $p < 0.0001$ for *Mki67*; $p < 0.0001$, $p = 0.0106$, $p < 0.0001$ for *Sox9*; $p = 0.0154$, $p < 0.0001$, $p = 0.0005$ for *Pgc*.

h Corpus organoids cultured with or without TNFa (100 ng/mL) and a NF-κB inhibitor QNZ (80 nM). Scale bar, 500 μm.

i qPCR analysis of gastric epithelial cell marker expression in the corpus organoids in (h). Data are presented as mean fold changes ± SD (n = 4 biologically independent samples). TNFa vs Control $p = 0.0002$, TNFa + QNZ vs Control $p = 0.0733$, TNFa + QNZ vs TNFa $p = 0.0049$ for *Muc5ac*; $p < 0.0001$, $p = 0.0038$, $p < 0.0001$ for *Gkn2*; $p = 0.0009$, *n.s.*, $p = 0.0002$ for *Aqp3*; $p = 0.0001$, $p = 0.0085$, $p < 0.0001$ for *Mki67*; *n.s.*, $p = 0.0004$, $p = 0.0012$ for *Sox9*; $p < 0.0001$, $p < 0.0001$, $p = 0.0036$ for *Pgc*.

Statistical information: significance was calculated by two-tailed Student's t-tests for samples with equal variances or two-sided Welch's t-tests for samples with unequal variances in (a), (b), (d). * $p < 0.05$; ** $p < 0.01$. In (g) and (i), significance was calculated by one-way ANOVA followed by Tukey post hoc test (significance level, $p < 0.05$). Source data are provided as a Source Data file.

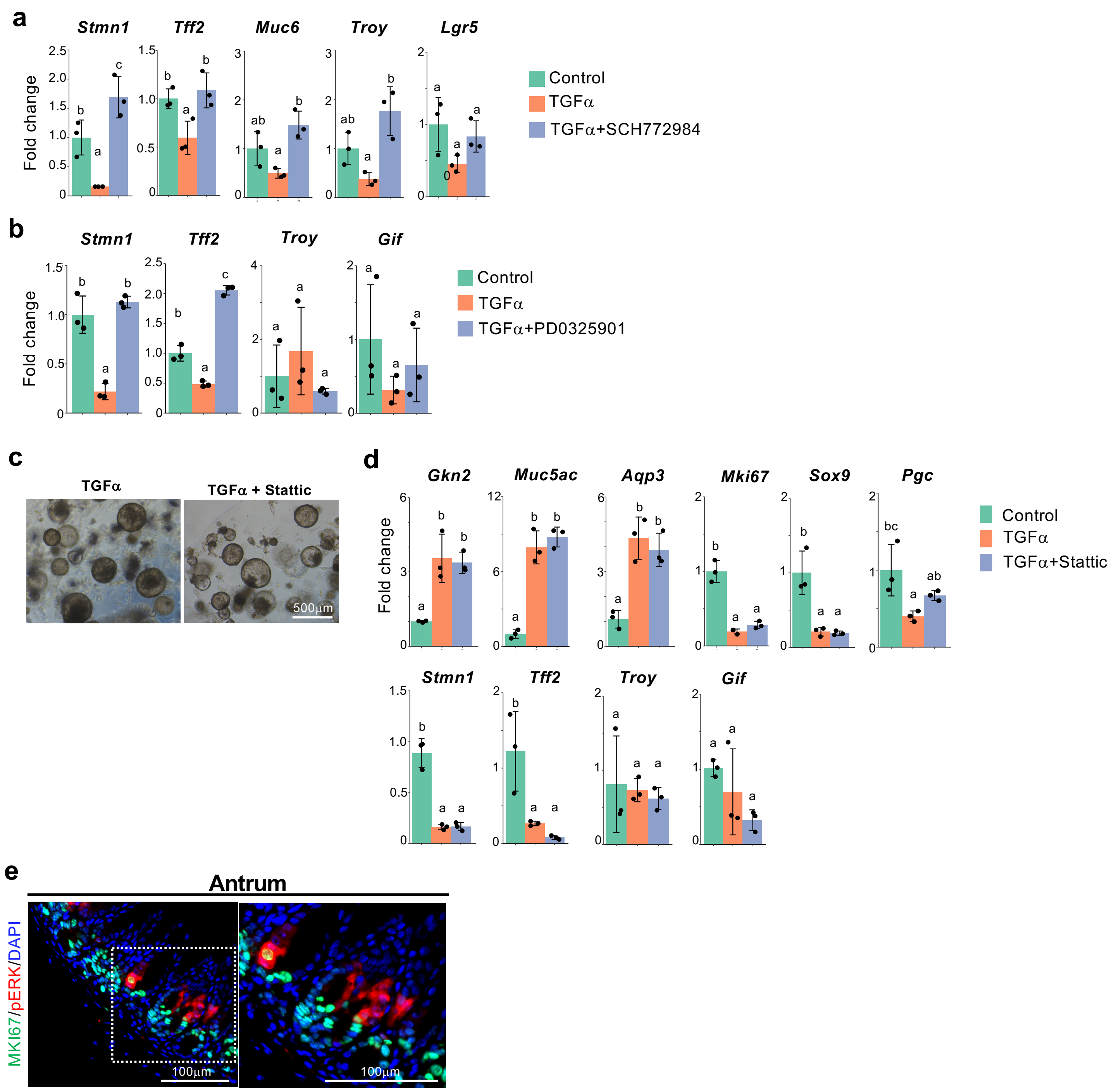

a
Stmn1
Tff2
Muc6
Troy
Lgr5
Fold change
Control
TGFα
TGFα+SCH772984
b
Stmn1
Tff2
Troy
Gif
Fold change
Control
TGFα
TGFα+PD0325901
c
TGFα
TGFα + Stattic
500µm
d
Gkn2
Muc5ac
Aqp3
Mki67
Sox9
Pgc
Fold change
Control
TGFα
TGFα+Stattic
Stmn1
Tff2
Troy
Gif
e
Antrum
MKI67/pERK/DAPI
100µm
100µm

Supplementary Fig. 25. Activation of ERK pathway is required for pit cell differentiation. Related to Fig. 7

a qPCR analysis of gastric epithelial cell markers in corpus organoids cultured with or without TGFα (12.5 ng/mL) and an ERK1/2 inhibitor, SCH772984 (0.4 μM). Data are presented as mean fold changes ± SD (n = 3 biologically independent samples). TGFα vs Control $p = 0.0197$, TGFα + SCH vs Control $p = 0.0449$, TGFα + SCH vs TGFα $p = 0.0010$ for *Stmn1*; $p = 0.0437$, *n.s.*, $p = 0.0196$ for *Tff2*; *n.s.*, *n.s.*, $p = 0.0072$ for *Troy*; *n.s.*, *n.s.*, *n.s.* for *Lgr5*.

b qPCR analysis of gastric epithelial cell markers in corpus organoids cultured with or without TGFα (12.5 ng/mL) and a MEK inhibitor PD0325901 (1 μM). Data are presented as mean fold changes ± SD (n = 3 biologically independent samples). TGFα vs Control $p = 0.0006$, TGFα + PD03 vs Control *n.s.*, TGFα + PD03 vs TGFα $p = 0.0002$ for *Stmn1*; $p = 0.0012$, $p < 0.0001$, $p < 0.0001$ for *Tff2*; *n.s.*, *n.s.*, *n.s.*, for *Troy*; *n.s.*, *n.s.*, *n.s.*, for *Gif.*

c Corpus organoids treated with or without a Stat3 inhibitor Stattic. The organoids were cultured in the presence of TGFα (12.5 ng/mL) with or without Stattic (50 μM) for 6 days.
Scale bar, 500 μm.

d qPCR analysis of gastric epithelial cell marker expression in the corpus organoids in (c). Data are presented as mean fold changes ± SD (n = 3 biologically independent samples for each culture condition). Note that Stattic did not affect the expression of pit cell markers and *Mki67*. TGFα vs Control $p = 0.0055$, TGFα + Stattic vs Control $p = 0.0079$, TGFα + Stattic vs TGFα *n.s.* for *Gkn2*; $p = 0.0002$, $p = 0.0001$, *n.s.* for *Muc5ac*; $p = 0.0027$, $p = 0.0047$, *n.s.* for *Aqp3*; $p < 0.0001$, $p = 0.0001$, *n.s.* for *Mki67*; $p = 0.0033$, $p = 0.0029$, *n.s.* for *Sox9*; $p = 0.0248$, *n.s.*, *n.s.* for *Pgc*; $p = 0.0001$, $p = 0.0001$, *n.s.* for *Stmn1*; $p = 0.0200$, $p = 0.0088$, *n.s.* for *Tff2*; *n.s.*, *n.s. n.s.* for *Troy*; *n.s.*, *n.s. n.s.* for *Gif.*

e Immunofluorescence staining of pERK (red), MKI67 (green), and DAPI (blue) in the mouse antrum tissue. Inset shows the high magnification image of boxed area. The image are a representative of three independent experiments.

Statistical information: significance was calculated by one-way ANOVA followed by Tukey post hoc test in (a), (b), and (d) (significance level, $p < 0.05$).

b-actin	F	GGCTGTATTCCCCTCCATCG
	R	CCAGTTGGTAACAATGCCATGT
Muc5ac	F	CAGGACTCTCTGAAATCGTACCA
	R	AAGGCTCGTACCACAGGGA
Gkn2	F	ATGAAACCCCTCGTGGCATTT
	R	TGTCTCCTGGATATTGCCTCC
Aqp3	F	GCTTTTGGCTTCGCTGTCAC
	R	TAGATGGGCAGCTTGATCCAG
Mki67	F	ATCATTGACCGCTCCTTTAGGT
	R	GCTCGCCTTGATGGTTCCT
Sox9	F	AGTACCCGCATCTGCACAAC
	R	ACGAAGGGTCTCTTCTCGCT
Pgc	F	CCACCTACTACACTCAAGGGC
	R	AACTCCTGGTTAGGGACCTGG
Stmn1	F	TCTGTCCCCGATTTCCCCC
	R	AGCTGCTTCAAGACTTCCGC
Tnfrsf12a	F	GTGTTGGGATTCGGCTTGGT
	R	GTCCATGCACTTGTCGAGGTC
Tff2	F	TGCTCTGGTAGAGGGCGAG
	R	CGACGCTAGAGTCAAAGCAG
Muc6	F	CGGCTGCGTCTGTCCTAAG
	R	GCATAGTCACATGGGCATTCCT
Gif	F	CCCTCTACCTCCTAAGTGTTCTC
	R	CTGAGTCAGTCACCGAGTTCT
Troy	F	GACTGCCTGCCAGGATTTTAC
	R	CAGTGTGGTTCGTAGGGAGG
Lgr5	F	CCTACTCGAAGACTTACCCAGT
	R	GCATTGGGGTGAATGATAGCA

Supplementary Table 1. List of the primers used in this study.
