# Supplementary material for: Single-cell transcriptomics uncovers EGFR signaling-mediated gastric progenitor cell differentiation in stomach homeostasis
Source: Nat Commun. 2023 Jun 29;14:3750. doi: 10.1038/s41467-023-39113-0 (PMC10310803; doi:10.1038/s41467-023-39113-0)
Supplement: Supplementary file 3 — Description of Additional Supplementary Files [file 41467_2023_39113_MOESM3_ESM.pdf]

Description of Additional Supplementary Files

Supplementary Data 1: List of pseudotime dependent genes along the differentiation trajectory from isthmus progenitor cells to pit cells in dataset 2

Supplementary Data 2: List of pseudotime dependent genes along the differentiation trajectory from isthmus progenitor cells to pit cells in dataset 1

Supplementary Data 3: List of pseudotime dependent genes along the differentiation trajectory from isthmus progenitor cells to neck cells in dataset 2

Supplementary Data 4: List of pseudotime dependent genes along the differentiation trajectory from isthmus progenitor cells to neck cells in dataset 1

Supplementary Data 5: List of pseudotime dependent genes along the differentiation trajectory from isthmus progenitor cells to parietal cells in dataset 2

Supplementary Data 6: List of the pseudotime dependent transcription factors in dataset 2

Supplementary Data 7: List of the pseudotime dependent transcription factors in dataset 1
