# Supplementary material for: Single-cell transcriptomics uncovers EGFR signaling-mediated gastric progenitor cell differentiation in stomach homeostasis
Source: Nat Commun. 2023 Jun 29;14:3750. doi: 10.1038/s41467-023-39113-0 (PMC10310803; doi:10.1038/s41467-023-39113-0)
Supplement: Supplementary file 11 — Reporting Summary [file 41467_2023_39113_MOESM11_ESM.pdf]

nature portfolio

Corresponding author(s): Akira Kurisaki, PhD

Last updated by author(s): Apr 26, 2023

Reporting Summary

Nature Portfolio wishes to improve the reproducibility of the work that we publish. This form provides structure for consistency and transparency in reporting. For further information on Nature Portfolio policies, see our Editorial Policies and the Editorial Policy Checklist.

Statistics

For all statistical analyses, confirm that the following items are present in the figure legend, table legend, main text, or Methods section.

n/a	Confirmed	
☐	☒	The exact sample size (*n*) for each experimental group/condition, given as a discrete number and unit of measurement
☐	☒	A statement on whether measurements were taken from distinct samples or whether the same sample was measured repeatedly
☐	☒	The statistical test(s) used AND whether they are one- or two-sided<br>*Only common tests should be described solely by name; describe more complex techniques in the Methods section.*
☒	☐	A description of all covariates tested
☐	☒	A description of any assumptions or corrections, such as tests of normality and adjustment for multiple comparisons
☐	☒	A full description of the statistical parameters including central tendency (e.g. means) or other basic estimates (e.g. regression coefficient) AND variation (e.g. standard deviation) or associated estimates of uncertainty (e.g. confidence intervals)
☐	☒	For null hypothesis testing, the test statistic (e.g. *F*, *t*, *r*) with confidence intervals, effect sizes, degrees of freedom and *P* value noted<br>*Give P values as exact values whenever suitable.*
☒	☐	For Bayesian analysis, information on the choice of priors and Markov chain Monte Carlo settings
☒	☐	For hierarchical and complex designs, identification of the appropriate level for tests and full reporting of outcomes
☒	☐	Estimates of effect sizes (e.g. Cohen's *d*, Pearson's *r*), indicating how they were calculated

Our web collection on statistics for biologists contains articles on many of the points above.

Software and code

Policy information about availability of computer code

Data collection

For immunofluorescence analysis, image acquisition was performed using KEYENCE microscope (BZ-X710) with BZ-X Viewer (01.03.02.01) or confocal laser microscope (LSM710) with ZEN 2009 software was used. For phase contrast images, image acquisition was performed using Leica DMIL LED with Leica MC120 HD.
Single-cell sorting was performed using MoFlo Astrios EQ Cell Sorter. The sequencing library was analyzed using the NextSeq 500/550 High Output v2 Kit (75 cycles) (Illumina).To obtain the digital expression matrix, fastq files were processed using Drop-seq tools (version 2.4.1), FASTX-Toolkit (version 0.0.14), STAR (version 2.7.8a.), and correct_bacode.py. The GRCm39 primary genome assembly reference genomes and Gencode Release M27 (GRCm39) were used.

Data analysis

For immunofluorescence analysis, Fiji software (Version: 2.1.0/1.53c, https://imagej.net/software/fiji/downloads) was used to quantify the fluorescence intensity and count the number of cells.
Single-cell RNA-seq data analysis was performed using Surat4 (version 4.1.0). A pseudotime analysis was performed using FateID (version 0.1.9). FateID software was installed in R directly from github (https://github.com/dgrun/FateID) using devtools (version 2.3.2; https://cran.r-project.org/web/packages/devtools).For hierarchical clustering of gene co-expression nodes, the R package pheatmap (version 1.0.12) was used to draw heatmaps of average expression levels of genes belonging to each node and to perform hierarchical clustering on a set of nodes. For the analysis of cell-cell communication, we used CellChat (version 1.4.0). R scripts for Seurat processing are available on GitHub [https://github.com/satijalab/Seurat]. StackedVlnPlot is available on [https://divingintogeneticsandgenomics.rbind.io/post/stacked-violin-plot-for-visualizing-single-cell-data-in-seurat/]. FateID for R is available on github [https://github.com/dgrun/FateID]. Devtools and pheatmap are available on [https://cran.r-project.org/web/packages/devtools] and [http://cran.r-project.org/web/packages/pheatmap], respectively.
Summit software (V6.3.0.16900) was used for flow cytometry data collection and data visualization.

For manuscripts utilizing custom algorithms or software that are central to the research but not yet described in published literature, software must be made available to editors and reviewers. We strongly encourage code deposition in a community repository (e.g. GitHub). See the Nature Portfolio guidelines for submitting code & software for further information.

Data

Policy information about availability of data

All manuscripts must include a data availability statement. This statement should provide the following information, where applicable:

- Accession codes, unique identifiers, or web links for publicly available datasets
- A description of any restrictions on data availability
- For clinical datasets or third party data, please ensure that the statement adheres to our policy

Raw data and digital expression matrix for the single-cell RNA-seq in this study have been deposited in Gene Expression Omnibus (GEO) under accession code GSE216139 [https://www.ncbi.nlm.nih.gov/geo/query/acc.cgi?acc=GSE216139]. Source data are provided with this paper.

Human research participants

Policy information about studies involving human research participants and Sex and Gender in Research.

Reporting on sex and gender: N/A

Population characteristics: N/A

Recruitment: N/A

Ethics oversight: N/A

Note that full information on the approval of the study protocol must also be provided in the manuscript.

Field-specific reporting

Please select the one below that is the best fit for your research. If you are not sure, read the appropriate sections before making your selection.

☒ Life sciences ☐ Behavioural & social sciences ☐ Ecological, evolutionary & environmental sciences

For a reference copy of the document with all sections, see nature.com/documents/nr-reporting-summary-flat.pdf

Life sciences study design

All studies must disclose on these points even when the disclosure is negative.

Sample size

For the single-cell RNA-sequencing, we did not perform sample size calculation. In both dataset 1 and 2, two biological replicates (each was derived from three mice) were prepared by two different operators. We confirmed that the biological replicate data overlapped nicely. Sample size calculation was not performed in advance. For organoid experiments and in vivo mouse experiments, sample size efficiency was based on previous studies in the same field (Cell Stem Cell Volume 26, Issue 6, June 2020, Pages 910-925). Sample sizes were indicated in each figure legend. The number of gastric units counted in each mice were also indicated in each figure legend.

Data exclusions

For the single-cell RNA-seq data analysis, if the detected gene count was less than 2500, the cell was defined as low-quality and excluded from further analyses.

Replication

Sample sizes were indicated in each figure legends, and we confirmed that any replicates were successful.

Randomization

For gastric organoid experiments, samples were based on culture conditions. For in vivo experiments, C57BL/6J mice were allocated to vehicle and erlotinib treatment groups. In all experiments, control and treatment group were analyzed in parallel.

Blinding	Blinding was not used in this study as single-cell RNA-seq analysis and qPCR analysis were not affected by investigator's expectations. For quantification of immunofluorescence images, the same thresholds were applied to control and treatment groups, and measurement was performed by Fiji software (version: 2.1.0).

Reporting for specific materials, systems and methods

We require information from authors about some types of materials, experimental systems and methods used in many studies. Here, indicate whether each material, system or method listed is relevant to your study. If you are not sure if a list item applies to your research, read the appropriate section before selecting a response.

Materials & experimental systems

n/a	Involved in the study
☐	☒ Antibodies
☒	☐ Eukaryotic cell lines
☒	☐ Palaeontology and archaeology
☐	☒ Animals and other organisms
☒	☐ Clinical data
☒	☐ Dual use research of concern

Methods

n/a	Involved in the study
☒	☐ ChIP-seq
☐	☒ Flow cytometry
☒	☐ MRI-based neuroimaging

Antibodies

Antibodies used

Primary antibodies:
AGR2 (Abcam, ab76473, 1:400)
ATP4B (ATLAS ANTIBODIES, HPA045400, 1:400)
AQP3 (Abcam, ab125219, 1:400)
BASP1 (Noves Biologicals, NBP1-68958, 1:1000)
CD4 (Santa Cruz, sc-13573, 1:100)
CD31 (BD Pharmingen, 557355, 1:100, 1:400)
Cleaved Caspase-3 (Cell Signaling, D175, 1:400)
EGFR (Abcam, ab52894, 1:400)
pERK (Cell Signaling, 4370S, 1:200)
GKN2 (Abcan, ab188866, 1:800)
IGF-1R (Cell Signaling, 3027S, 1:200)
KRT20 (Proteintech, 17329-1-AP, 1:400)
KRT7 (Abcam, ab181598, 1:400)
MKI67 (Abcam, ab15580, 1:400)
MKI67 (ThermoFisher, 14-5698-82, 1:1000)
NFKB1 (Abcam, ab32360, 1:400)
PDGFRa (Invitrogen, 14-1401-81, 1:200)
PGC (Abcam, ab31464, 1:2000)
SMA (Novus Biologicals, NBP1-30894, 1;400)
Somatostatin (Santa Cruz, sc-7819, 1:800)
SOX9 (Abcam, ab185230, 1:400)

Secondary antibodies:
Donkey anti-Rat IgG (H+L) Alexa Fluor 488 (Invitrogen, A-21208, 1:1000)
Donkey anti Rabbit IgG (H+L) Alexa Fluor 488 (Invitrogen, A-21206, 1:1000)
Donkey anti Rabbit IgG (H+L) Alexa Fluor 555 (Invitrogen, A-31572, 1:1000)
Donkey anti-Sheep IgG (H+L) Alexa Fluor 594 (Invirogen, A-11016, 1:1000)
Anti-IgG (H+L chain) (Rabbit) pAb-HRP (MBL, 458, 1:10,000)

Lectin GS-II Griffonia simplicifolia, Alexa Fluor 647 conjugate (Invitrogen, L32451, 1:400)

Validation

All primary antibodies were validated by manufacturers at least by Western Blotting. We also tested the specificity of antibodies using the samples with no primary antibodies in the initial validation. For immunofluorescence analysis of organoid experiments, stomach tissue samples were included as positive controls.

Animals and other research organisms

Policy information about studies involving animals; ARRIVE guidelines recommended for reporting animal research, and Sex and Gender in Research

Laboratory animals

All mice were fed ad libitum and housed in a specific pathogen-free facility under the following conditions: a 12 h light-dark cycle, ambient temperature of 23 to 24°C, and 50 to 70% humidity. C57BL/6J mice at 10 weeks old were used for single-cell RNA-sequencing. C57BL/6J mice at 7-10 weeks old were used for in vivo erlotinib treatment.

Wild animals	No wild animals were used in this study.
Reporting on sex	Sex were not considered in this study design. Male mice were used for single-cell RNA sequencing. Female mice were used for in vivo erlotinib treatment.
Field-collected samples	No field-collected samples were used in this study.
Ethics oversight	All animal experiments were performed according to the procedures approved by the animal welfare and ethical review panel of Nara Institute of Science and Technology and RIKEN.

Note that full information on the approval of the study protocol must also be provided in the manuscript.

Flow Cytometry

Plots

Confirm that:

☒ The axis labels state the marker and fluorochrome used (e.g. CD4-FITC).

☒ The axis scales are clearly visible. Include numbers along axes only for bottom left plot of group (a 'group' is an analysis of identical markers).

☒ All plots are contour plots with outliers or pseudocolor plots.

☒ A numerical value for number of cells or percentage (with statistics) is provided.

Methodology

Sample preparation	In dataset 1, dissociated cells resuspended in 0.1% BSA/PBS were stained with PI and used for single-cell sorting. In dataset 2, dissociated cells were stained with Hoechst 33432 and TO-PRO-3 and used for single-cell sorting. The detailed cell dissociation protocols were indicated in Methods section.
Instrument	Single-cell sorting was performed by MoFlo Astrios EQ Cell Sorter (Beckman Coulter, Brea, CA, USA).
Software	Summit software (V6.3.0.16900) was used for flow cytometry data collection and data visualization.
Cell population abundance	In Dataset 1, the purity of the gate fraction used for one-cell sorting was 21.93% of the total event; in Dataset 2, it was 26.38%. The gate specification is described in the "Gating strategies" section below.
Gating strategy	We showed the detail of gating strategies in SUPPLEMENTARY FIGURE 1. In Dataset 1, the following steps were performed for the gating. First, a region (R1) was selected using information from FSC and SSC, excluding events with very low signal intensity. Based on the boundary area that is the valley of the bimodal population in the fluorescence intensity of PI dye, the population with relatively low intensity was designated as a PI negative region (R7), and a viable cell gate (R1&R7) was selected. Finally, a singlet gate (R1&R7&R9) was selected based on FSC information (Area/Width). The selected singlet gate was examined by a microscope to evaluate whether sorted cells were really singlets or not. In Dataset 2, the following flow was used for gating. The fluorescence intensity of TO-PRO-3 dye was bimodal, and the population with relatively low intensity was selected as a viable cell region (R9) using the valley between the bimodal peaks as the boundary line. The cell population with nuclei (R7&R9) was selected using the fluorescence intensity of Hoechst 33342 dye and FSC information. Finally, a singlet gate (R5&R7&R9) was selected using FSC information (Area/Height). The selected singlet gate was examined microscopically by sorting cells to see if it was indeed singlet gate.

☒ Tick this box to confirm that a figure exemplifying the gating strategy is provided in the Supplementary Information.
